# A new method for a priori practical identifiability

**DOI:** 10.1371/journal.pone.0327593

**Published:** 2025-07-17

**Authors:** Peter Thompson, Benjamin Jan Andersson, Nicolas Sundqvist, Gunnar Cedersund

**Affiliations:** Department of Biomedical Engineering Linköping University, Linköping, Sweden; Minnan Normal University, CHINA

## Abstract

*Background and objective*: Practical identifiability analysis, i.e., ascertaining whether a model property can be determined from given data, is central to model-based data analysis in biomedicine. The main approaches used today all require that coverage of the parameter space be exhaustive, which is usually impossible. An alternative could be using structural identifiability methods, since they do not need such coverage. However, current structural methods are unsuited for practical identifiability analysis, since they assume that all higher-order derivatives of the measured variables are available. Herein, we provide new definitions and methods that allow for this assumption to be relaxed. *Methods and results*: We introduce the concept of $\left(\nu_{1},...,\nu_{m}\right)$-identifiability, which differs from previous definitions in that it assumes that only the first $\nu_{i}$ derivatives of the measurement signal *y*_*i*_ are available. This new type of identifiability can be determined using our new algorithms, as is demonstrated by applications to various published biomedical models. Our methods allow for identifiability of not only parameters, but of any model property, i.e., observability. These new results provide further strengthening of conclusions made in previous analysis of these models. For the first time, we can quantify the impact of the assumption that all derivatives are available in specific examples. If one, e.g., assumes that only up to third order derivatives, instead of all derivatives, are available, the number of identifiable parameters drops from 17 to 1 for a Drosophila model, and from 21 to 6 for an NF-κB model. In both models, the previously obtained identifiability is present only if at least 20 derivatives of all measurement signals are available. *Conclusion*: Our results demonstrate that the assumption regarding availability of derivatives made in traditional structural identifiability analysis requires a big overestimation regarding the number of parameters that can be estimated. Our new methods and algorithms allow for this assumption to be relaxed, bringing structural identifiability methodology one step closer to practical identifiability analysis.

## 1 Background

Biology and medicine involve the study of a myriad of time-dependent variables, which cross-talk with each other in complex networks. To deal with this complexity, one often makes use of mechanistic mathematical models, often formulated using ordinary differential equations (ODEs). These ODEs represent hypotheses regarding the biological mechanisms that can explain given biological data [9]. The model-based data analysis of such real data revolves around a correct uncertainty analysis of parameters and predictions: to what degree can one determine the parameters and predictions of interest? This uncertainty analysis is formally called identifiability analysis if it deals with parameter uncertainty, and observability analysis if it deals with other model properties such as states and composite variables [10]. Both observability and identifiability analysis can be done in many different ways, but they are generally subdivided into two types of approaches: structural and practical.

Practical identifiability analysis revolves around the specific data one has collected in a specific situation [10]. In other words, practical identifiability analysis looks at the real situation, where the data has noise, and where the experiment might not have been done in an optimal way to generate information about all parameters. Historically, one has often used sensitivity-based methods to study practical identifiability, such as analysis of the Fisher Information Matrix [35], and the covariance of the parameters [11]. In the last 10 years, these methods have been increasingly replaced by Markov Chain Monte Carlo (MCMC) and profile-likelihood based methods [10,11,25]. The benefit of these more modern methods is that they study global properties, and do not have to assume that the system is identifiable to assess identifiability [10,35]. These methods are highly useful and flexible, but they still have limitations. Most importantly, they rely on the exhaustive coverage of the entire parameter space, either using the MCMC sampling or using the optimization step in profile likelihood. For large models, this optimization step is difficult and time-consuming even for an expert in the field [26,30]. This difficulty will always leave the analysis results with an inherent doubt: if the coverage of the parameter space fails, the degree of identifiability (the calculated confidence intervals) is not correct.

Structural identifiability analysis has historically been the mirror image of practical identifiability analysis. Structural methods normally do not take a specific data set or the noise level into account. Instead, structural identifiability analysis only considers the form of the equations, including the measurement equations. Furthermore, structural identifiability analysis does not rely on an exhaustive coverage of the entire parameter space. Instead, structural identifiability analysis makes use of powerful theories from differential algebra and differential geometry to investigate whether there are structural limits for whether a parameter or a model property ever can be determined from a given measurement. Methods have been developed to determine which states and parameters are locally identifiable [29] and globally identifiable [6,15], to discover parameter combinations that are identifiable [19,20,23,24,30], and to suggest modifications in the modeling process based on identifiability [4,14]. In the last two decades, powerful methods have been developed that can do such an analysis also for relatively large biological models, featuring 50 to 100 states and parameters [4].

Because structural identifiability methods now can be applied to real, large models, it would be highly interesting if they also could perform practical identifiability analysis, where the limitations of a specific dataset are taken into account. One prominent such assumption is that current methods assume that all derivatives of all measurement signals can be estimated from the data. In practical situations, with real data, this assumption is never fulfilled. It is therefore a critical flaw that we are lacking definitions and methods to analyze the consequences of this unfulfilled assumption.

In summary, there is a need to develop new methods that combine the strengths of the two approaches: a method that does not rely on overly idealistic assumptions regarding the real situations, but that also does not rely on optimization and coverage of the entire parameter space. However, such methods have not yet been proposed. Herein, we introduce a new type of practical *and* structural identifiability, which puts an upper limit on how many times each measurement signal can be differentiated. We also introduce new algorithms that can calculate this new type of identifiability, and apply these to a series of published models. For all studied examples, traditional structural identifiability methods have widely overestimated the number of identifiable parameters: with a maximum of 3 derivatives available, more than 90% of the previously identifiable parameters lose their identifiability.

We present a new method for determining structural identifiability in a more realistic setting, where only a given finite number of derivatives are available for each measurement signal. While this is novel, there are a few related methods that should be mentioned. Most structural identifiability methods assume that all derivatives of outputs are measurable and all derivatives of all outputs are sufficiently varying. The latter condition is not always satisfied in practical modeling as, for example, a constant function may be used as an input, and this may lead to incorrect implementation of a model. In [32] a method for determining how many derivatives of the inputs must be non-zero was given, and an implementation was presented in [33]. In [4] an implementation was given for determining minimal sets of outputs that make a system structurally locally identifiable.

## 2 Results and discussion

### 2.1 Problem: There is an upper limit for how many derivatives can be estimated from biological data

There is usually an upper limit for the number of derivatives of an output signal that can be estimated from data. This is illustrated in the following example.

**Example 2.1.** *Consider the system*


$$x_{1}^{\prime }=\theta x_{2},\;x_{2}^{\prime }=-\theta x_{1}+1,\;y=x_{1}$$

**Fig 1:**
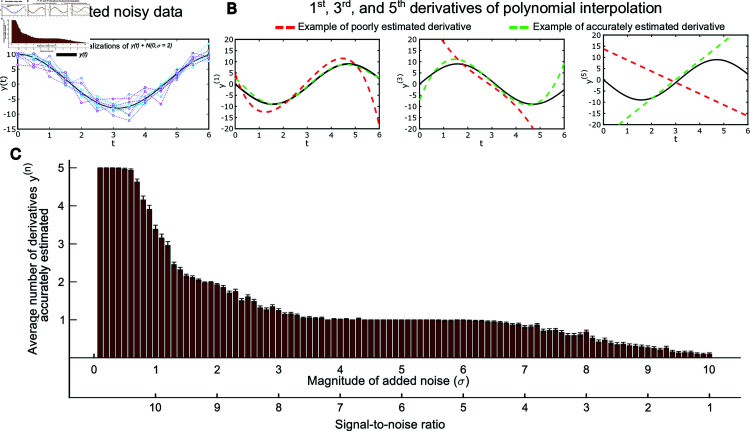
Illustration of the upper limit for how many derivatives can be estimated from noisy data. **A:** Examples of 10 different realizations of noisy data (colored lines) and the true function y(t)=1+9cos(t) (black line) **B:** Illustrations of the $1^{\text{st}}$, $3^{\text{rd}}$, and $5^{\text{th}}$ derivatives of the function *y*(*t*) with examples of a poorly estimated derivative (red line) and an accurately estimated derivative (green line). **C:** The relationship between the average number of accurately estimated derivatives and the magnitude of the added noise σ/the signal-to-noise ratio. The bars show the mean number of accurately estimated derivatives at each noise level and the error bars show the standard error of the mean (SEM).

*where x_1_ and x_2_ are state variables, θ is a constant parameter, and y is an observable output variable; that is y is measurable by experiments but x_1_, x_2_ and θ are unknown. Suppose that, unbeknownst to the observer, θ=1, x_1_(0) = 10, and x_2_(0) = 0. Consequently the true signal of y is given by y(t)=1+9cos(t). However, in practice, the measured signal will not show a smooth curve but rather a discrete time series that also has added noise. An illustration of this measurement signal can be found in Fig 1A, where 10 realizations of the function y(t)+N(0,σ=2), where the second term represents normally distributed noise with mean 0 and standard deviation 2, are depicted alongside the true function y(t) (Fig 1A). The number of derivatives that can accurately be estimated can be evaluated by deriving the function of a polynomial interpolation of these data points and comparing these estimated derivatives to the derivatives of the true function (Fig 1B). More specifically, this example shows a 6^*th*^ degree polynomial interpolation for t-values ranging from 0 to 6 in increments of 0.5. Subsequently, the 0^*th*^ to the 5^*th*^ derivatives of these interpolations are then compared to the derivatives of the true function y(t). The number of derivatives that can accurately be estimated is dependent on the magnitude of noise in the system. More specifically, the magnitude of the added noise increases the number of accurately estimated derivatives declines (Fig 1C). To illustrate this dependency, the example above was repeated with the added noise sigma ranging from 0.1 to 10 in increments of 0.1 (σ=[0.1,0.2,…,10]) and with 1000 data realizations generated at each noise level. Fig 1C shows the average number of derivatives that could accurately be estimated at each noise level. A derivative is considered accurate if the square root of the sum of squared residuals does not exceed the amplitude of y. From this figure, it is clear that the number of derivatives that can accurately be estimated diminishes rapidly as the signal-to-noise ratio approaches 1.*

The following example illustrates how this lack of availability of all derivatives of *y* is associated with an unrealistic assessment of whether parameters are identifiable.

**Example 2.2.** *Consider the system*


$$\left\{ \begin{array}{c} x_{1}^{\prime }=\theta_{1}x_{2}\\ x_{2}^{\prime }=-\theta_{1}x_{1}\\ x_{3}^{\prime }=\theta_{1}\theta_{2}\\ y=x_{1}+x_{3}, \end{array}\right.$$

*where x_1_, x_2_, and x_3_ are (unobserved) state variables, $\theta_{1}$ and $\theta_{2}$ are constant parameters, and y is an (observed) output variable.*

*Running the algorithm from [29] shows that x_1_, x_2_, x_3_, $\theta_{1}$, and $\theta_{2}$ are locally identifiable. Let us attempt to estimate $\theta_{1}$ from observation of y. A relation among $\theta_{1}$ and y over ℂ is $\theta_{1}^{2}y^{\prime \prime }+y^{\left(4\right)}=0$. Thus we can estimate $\theta_{1}$ by using our observed values of $y^{\prime \prime }$ and ${\text{y}}^{\left(4\right)}$ at some time t_0_:*


$$\theta_{1}=\pm \sqrt{\frac{-y^{\left(4\right)}(t_{0})}{y^{\prime \prime }(t_{0})}}.$$

*In practice, the measured signal y may be too noisy to give an accurate estimation of ${\text{y}}^{\left(4\right)}$. In such a situation, $\theta_{1}$ is not identifiable from the given data, even though a structural analysis says that it is. Suppose now that we know that our measurements are good enough to give reliable estimates of $y^{\prime \prime }$ but of no higher derivative of y. To use structural methods to determine the degree of identifiability in such a situation, we need new definitions and methods.*

**Remark 2.3.** *Our approach assumes measurements of several derivatives of the output signal are made at a single time. In practice it is more common to measure the 0-th derivative of the output signal at several times. Having measurements with some uncertainty of $y(t_{0}),\ldots ,y(t_{N})$ is equivalent to having measurements with some uncertainty of $y(t_{0}),\ldots ,y^{\left(N\right)}(t_{0})$. Hence this aspect of our approach does not limit its generality.*

*For Example 2.2, one can estimate $\theta_{1}$ using the relation $y^{\prime \prime }+\theta_{1}^{2}y-\theta_{1}^{3}\theta_{2}t-\theta_{1}^{2}x_{3}(0)=0$ together with measurements of y and $y^{\prime \prime }$ at three different times. However knowledge of $y,y^{\prime },y^{\prime \prime }$ at three times can be used to estimate $y,y^{\prime },y^{\prime \prime },y^{\left(3\right)},y^{\left(4\right)}$ at a single time.*

### 2.2 A new definition for practical and structural identifiability: $\left(\nu_{1},\ldots ,\nu_{m}\right)$-identifiable

We now provide an intuitively straightforward definition that is useful for understanding the new methods. In Sect 5, we provide a precise definition, together with analytical proofs of all stated method properties.

Let ℓ be a non-negative integer and let *n*, *m*, and *r* be positive integers. Let $\theta =(\theta_{1},\ldots ,\theta_{\ell })$, $𝐱=(x_{1},\ldots ,x_{n})$, $𝐲=(y_{1},\ldots ,y_{m})$, and $𝐮=(u_{1},\ldots ,u_{r})$. Let $𝐟=(f_{1},\ldots ,f_{n})$ and $𝐠=(g_{1},\ldots ,g_{m})$ be tuples of rational functions in **x**, **u**, and θ over ℂ. This setup determines a class of systems of ODEs with initial conditions, or a model class:

$$\Sigma ({𝐱}^{*},\theta ,𝐮)=\left\{ \begin{array}{c} \frac{d𝐱}{dt}=𝐟(𝐱,\theta ,𝐮),\\ 𝐲=𝐠(𝐱,\theta ,𝐮),\\ 𝐱(0)={𝐱}^{*}, \end{array}\right.$$ (1)

where **x** are state variables, which usually correspond to time-varying concentrations or physical properties; $x^{*}$ are the initial values of the state variables; θ are constant parameters, e.g. corresponding to rate constants or volumes; **u** are input variables, which are observed and typically controlled by the experimentalist; and **y** are output variables, which are observed experimentally. With these notations in place, the new definition is roughly given by:

**Definition 2.4** (roughly stated). *Let $\nu_{1},\ldots ,\nu_{m}$ be non-negative integers. Rational expression h∈ℂ(𝐱,θ) is said to be $\left(\nu_{1},\ldots ,\nu_{m}\right)$-identifiable if perfect knowledge of the first $\nu_{i}$ derivatives of y_i_ at a particular time allows us to determine the value of h at that point in time, up to a finite set.*

This definition, which is more technically stated in Definition 5.4, provides the essence of the new definition: given only a limited set of derivatives of the different measurement signals, can we determine the parameter $\theta_{i}$ to be locally identifiable, i.e. as belonging to a finite set? This definition is analogous to traditional definitions of structural identifiability, with the only addition being that we now only have access to a finite set of derivatives. In other words, in traditional structural identifiability analysis, no such constraints on the number of derivatives available is present. This new definition is closer to practical identifiability since, with real biological data, one can only estimate a few derivatives from the measurement data. Note that in practice it is more common to measure the signal at multiple times. We explain in Remark 2.3 that this does not limit the generality of our approach.

### 2.3 Solution: Our new algorithms for calculating practical structural identifiability

For most models, it would be difficult to prove the $\left(\nu_{1},\ldots ,\nu_{m}\right)$-identifiability of a given parameter directly from the definition. We provide Algorithm 1, which can do this using only straightforward algebraic computations. The algorithm applies to any rational combination of states and parameters, of which an individual parameter is a special case.

**Algorithm 1 Determine whether a given element of ℂ(𝐱,θ) is ν-identifiable.**

**Input** : Equations ${𝐱}^{\prime }=𝐟(𝐱,\theta ,𝐮),\;𝐲=𝐠(𝐱,\theta ,𝐮)$

non-negative integers $\nu_{1},\ldots ,\nu_{m}$

rational expression h∈ℂ(𝐱,θ)

**Output** : “Yes” if *h* is $\left(\nu_{1},\ldots ,\nu_{m}\right)$-identifiable

“No” if *h* is not $\left(\nu_{1},\ldots ,\nu_{m}\right)$-identifiable Step 1: Write the matrix


$$J:=(\begin{array}{cccccc} \frac{\partial y_{1}}{\partial x_{1}} & \ldots & \frac{\partial y_{1}}{\partial x_{n}} & \frac{\partial y_{1}}{\partial \theta_{1}} & \ldots & \frac{\partial y_{1}}{\partial \theta_{\ell }}\\ \frac{\partial y_{1}^{\prime }}{\partial x_{1}} & \ldots & \frac{\partial y_{1}^{\prime }}{\partial x_{n}} & \frac{\partial y_{1}^{\prime }}{\partial \theta_{1}} & \ldots & \frac{\partial y_{1}^{\prime }}{\partial \theta_{\ell }}\\ \vdots & & \vdots & \vdots & & \vdots \\ \frac{\partial y_{1}^{\left(\nu_{1}\right)}}{\partial x_{1}} & \ldots & \frac{\partial y_{1}^{\left(\nu_{1}\right)}}{\partial x_{n}} & \frac{\partial y_{1}^{\left(\nu_{1}\right)}}{\partial \theta_{1}} & \ldots & \frac{\partial y_{1}^{\left(\nu_{1}\right)}}{\partial \theta_{\ell }}\\ \vdots & & \vdots & \vdots & & \vdots \\ \frac{\partial y_{m}}{\partial x_{1}} & \ldots & \frac{\partial y_{m}}{\partial x_{n}} & \frac{\partial y_{m}}{\partial \theta_{1}} & \ldots & \frac{\partial y_{m}}{\partial \theta_{\ell }}\\ \vdots & & \vdots & \vdots & & \vdots \\ \frac{\partial y_{m}^{\left(\nu_{m}\right)}}{\partial x_{1}} & \ldots & \frac{\partial y_{m}^{\left(\nu_{m}\right)}}{\partial x_{n}} & \frac{\partial y_{m}^{\left(\nu_{m}\right)}}{\partial \theta_{1}} & \ldots & \frac{\partial y_{m}^{\left(\nu_{m}\right)}}{\partial \theta_{\ell }} \end{array}).$$

Step 2: Compute the rank of *J*.

Step 3: Write the matrix *J*_*h*_, obtained by adding the row


$$\left(\begin{array}{cccccc} \frac{\partial h}{\partial x_{1}} & \ldots & \frac{\partial h}{\partial x_{n}} & \frac{\partial h}{\partial \theta_{1}} & \ldots & \frac{\partial h}{\partial \theta_{\ell }} \end{array}\right)$$

to *J*. Then calculate the rank of *J*_*h*_.

If $\text{rank}(J_{h})>\text{rank}(J)$, conclude that *h* is not $\left(\nu_{1},\ldots ,\nu_{m}\right)$-identifiable. (Output “No”).

If $\text{rank}(J_{h})=\text{rank}(J)$, conclude that *h* is $\left(\nu_{1},\ldots ,\nu_{m}\right)$-identifiable. (Output “Yes”).

In the case where one is interested in just an individual parameter or state, one can perform Algorithm 2, where one column is removed instead of a row added, resulting in a smaller matrix.

**Algorithm 2 Determine whether a given element of 𝐱∪θ is ν-identifiable.**

**Input** : Equations ${𝐱}^{\prime }=𝐟(𝐱,\theta ,𝐮),\;𝐲=𝐠(𝐱,\theta ,𝐮)$

non-negative integers $\nu_{1},\ldots ,\nu_{m}$

choice of $\theta_{i}\in \theta$ (resp. $x_{i}\in 𝐱$)

**Output** : “Yes” if $\theta_{i}$ (resp. *x*_*i*_) is $\left(\nu_{1},\ldots ,\nu_{m}\right)$-identifiable

“No” if $\theta_{i}$ (resp. *x*_*i*_) is not $\left(\nu_{1},\ldots ,\nu_{m}\right)$-identifiable Step 1: Write the matrix *J* as in Algorithm 1.

Step 2: Compute the rank of *J*.

Step 3: Remove the column corresponding to $\theta_{i}$ (resp. *x*_*i*_) and compute the rank of the resulting matrix.

If the rank has decreased, conclude that $\theta_{i}$ (resp. *x*_*i*_) is $\left(\nu_{1},\ldots ,\nu_{m}\right)$-identifiable. (Output “Yes”)

If the rank is the same, conclude that $\theta_{i}$ (resp. *x*_*i*_) is not $\left(\nu_{1},\ldots ,\nu_{m}\right)$-identifiable. (Output “No”)

The precise definition of ν-identifiability is given in Definition 5.4, and we prove that Algorithm 1 and 2 are correct in Sect 5.2.

The symbolic calculations can be impractically slow for all but the smallest models. Therefore we have also provided probabilistic analogs of these algorithms that are much faster in practice. These are presented and their correctness is proven in Sect 5.3.

### 2.4 Intuitive understanding of the new algorithms

We first discuss Algorithm 1. Each row of *J* represents how an output, or one of its derivatives, varies with respect to small changes in each state and parameter. If the last row of *J*_*h*_ is a linear combination of the other rows, then the variation of *h* can be accounted for as a combination of the variations of the output derivatives. This, in turn, means that it seems like *h* can be expressed in terms of the known signals, i.e. that *h* is identifiable. Conversely, if the last row of *J*_*h*_ is linearly independent of the other rows, then the variation of *h* cannot be explained in terms of that of the available derivatives of the outputs, and one should not expect to be able to determine *h* from the available outputs and their derivatives.

A similar explanation lies behind Algorithm 2. The column corresponding to $\theta_{i}$ is dependent on the other columns if and only if the variation in the outputs and their derivatives is fully explained by variation in the other parameters and states, which is true if and only if $\theta_{i}$ cannot be determined from numerical knowledge of the outputs and their derivatives, i.e. $\theta_{i}$ is not identifiable.

### 2.5 Examples

#### 2.5.1 Algorithm 1 determines identifiability of rational quantities, which is important in hypothesis testing.

Algorithm 1 (and its faster probabilistic version Algorithm 3) can be used to analyze so-called core predictions (see [10]). A core prediction is a well-determined property, i.e. a model prediction with a small uncertainty. Such core predictions are therefore often tested in future experiments, and are a central part of model-based hypothesis testing. Algorithm 1 can test whether or not such a model property really can be well-determined, given the available data. We illustrate this new possibility in the following two examples.

**Example 2.5.** *The following equations give a model of insulin (ins) binding and activation of insulin receptor (IR). This model was originally presented in [7], and is explained more in detail therein and in the supplementary materials.*


$$IR^{\prime }=-k_{1}\cdot IR\cdot u-k_{1,basal}\cdot IR+k_{R}\cdot IR_{i}+IR_{ins}\cdot k_{m,1}+k_{m,2}\cdot IR_{P},$$

$$IR_{ins}^{\prime }=k_{1}\cdot IR\cdot u-k_{2}\cdot IR_{ins}-k_{m,1}\cdot IR_{ins},$$

$$IR_{P}^{\prime }=k_{2}\cdot IR_{ins}+k_{1,basal}\cdot IR-k_{3}\cdot IR_{P}+k_{m,3}\cdot IR_{iP}-k_{m,2}\cdot IR_{P},$$

$$IR_{iP}^{\prime }=k_{3}\cdot IR_{P}-k_{m,3}\cdot IR_{iP}-k_{D}\cdot IR_{iP},$$

$$IR_{i}^{\prime }=k_{D}\cdot IR_{iP}-k_{R}\cdot IR_{i},$$

$$IRS^{\prime }=-k_{4}\cdot (IR_{P}+IR_{iP})\cdot IRS+k_{m,4}\cdot IRS_{P},$$

$$IRS_{P}^{\prime }=k_{4}\cdot (IR_{P}+IR_{iP})\cdot IRS-k_{m,4}\cdot IRS_{P},$$

$$y_{A}=k_{Y,A}\cdot (IR_{P}+IR_{iP}),$$

$$y_{B}=k_{Y,B}\cdot IRS_{P},$$

$$y_{C}=k_{Y,C}\cdot IRS_{P},$$

$$y_{D}=k_{Y,D}\cdot IRS_{P}$$

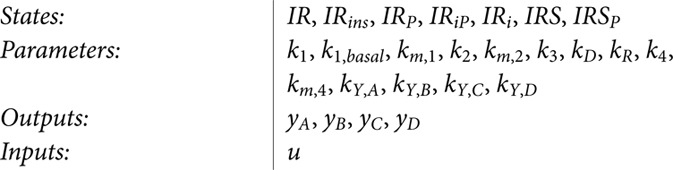



*This model was one of several hypotheses tested in the article. To draw one of the conclusions in that article, the authors looked at the proportion of insulin receptor bound to an internal membrane:*


$$prop_{i}:=\frac{IR_{i}}{IR+IR_{ins}+IR_{P}+IR_{iP}+IR_{i}}.$$

*It was predicted that prop_i_ should be between .55 and .80. Despite the fact that there are four outputs, none of the seven states (insulin receptor or substrate quantities at different locations) are identifiable, so it is not immediately obvious whether prop_i_ can be estimated from the measurements. Algorithm 3 with $\nu_{i}=n+\ell -1$ for all i shows that this quantity is indeed single-experiment locally identifiable (see Definition 5.8 and Corollary 5.10). The authors were able to experimentally determine the proportion to be 0.023±0.008. Thus they were able to reject the model, which was the conclusion in that step in the paper.*

*In summary, our analysis shows that the proportion of receptors that are bound to an internal membrane indeed is an identifiable model property. This new result holds from a structural point of view, without assuming that all derivatives are known. These results also show this without relying on optimization or exhaustive parameter sampling, as is done using the traditional practical identifiability analysis done in the original papers [7,10]. This new result thus strengthens and confirms the conclusion drawn in the original paper.*

**Example 2.6.** *The equations below constitute the model of the interaction between liver and pancreatic cells from [8].*


$$x_{1}^{\prime }=\frac{247x_{2}}{250}-\frac{247x_{1}}{250}+\frac{17\theta_{1}}{5000000}-\frac{17\theta_{2}x_{1}}{1500}-\frac{340\theta_{3}x_{1}x_{4}}{9}\left(1-\frac{\theta_{6}x_{5}}{\theta_{7}+x_{5}}\right),$$

$$x_{2}^{\prime }=\frac{247x_{1}}{250}-\frac{247x_{2}}{250},$$

$$x_{3}^{\prime }=\frac{247x_{4}}{250}-\frac{247x_{3}}{250}+\frac{100000000x_{7}\theta_{4}\theta_{8}x_{2}^{2}}{\frac{3537}{50}+100000000x_{2}^{2}},$$

$$x_{4}^{\prime }=\frac{247x_{3}}{250}-\frac{247x_{4}}{250}-\frac{17}{1500}\theta_{5}x_{4},$$

$$x_{5}^{\prime }=\frac{10000x_{1}}{3}-\frac{11}{2},$$

$$x_{6}^{\prime }=\frac{20x_{2}}{3}-\frac{x_{6}}{500},$$

$$x_{7}^{\prime }=\left(-\frac{81}{2500000}x_{6}^{2}+\frac{63}{100000}x_{6}-\frac{1}{400}\right)\theta_{9}x_{7},$$

$$y_{1}=\frac{5000}{3}(x_{1}+x_{2}),$$

$$y_{2}=\frac{5000}{3}(x_{4}+x_{3})$$

*There are seven states, $x_{1},\ldots ,x_{7}$, two outputs, $y_{1},y_{2}$, and no inputs. Numerical values were inserted for parameters whose values were known from operating specifications or estimated from the literature. The remaining parameters, here denoted by $\theta_{1},\ldots ,\theta_{9}$, were to be determined from experimental data. One quantity of interest was*


$$\theta_{3}\left(1-\frac{\theta_{6}x_{5}}{\theta_{7}+x_{5}}\right),$$

*representing hepatic insulin sensitivity (see [8, Fig. 4 Plot E]). Algorithm 3 tells us that this quantity is indeed identifiable. Moreover, by using different values of $\left(\nu_{1},\nu_{2}\right)$ we find that it is (6,5)-identifiable.*

#### 2.5.2 Identifiability tends to increase with increased order of observability.

It is typically the case that as we increase the number of derivatives that can be reliably estimated, the number of identifiable parameters increases, often dramatically. Therefore assuming that all derivatives are available can give a large overestimate of the number of identifiable parameters. How large this overestimate is can now be determined by our new algorithms. We illustrate this capability in the following two examples.

**Example 2.7** (Drosophila period protein). *The following equations constitute the model that was used in [13] to model Drosophila period protein.*


$$M^{\prime }=\frac{v_{s}K_{I}^{4}}{K_{I}^{4}+P_{N}^{4}}-\frac{v_{m}M}{K_{m}+M},$$

$$P_{0}^{\prime }=k_{s}M-\frac{V_{1}P_{0}}{K_{1}+P_{0}}+\frac{V_{2}P_{1}}{K_{2}+P_{1}},$$

$$P_{1}^{\prime }=\frac{V_{1}P_{0}}{K_{1}+P_{0}}+\frac{V_{4}P_{2}}{K_{4}+P_{2}}-P_{1}\left(\frac{V_{2}}{K_{2}+P_{1}}+\frac{V_{3}}{K_{3}+P_{1}}\right),$$

$$P_{2}^{\prime }=\frac{V_{3}P_{1}}{K_{3}+P_{1}}-P_{2}\left(\frac{V_{4}}{K_{4}+P_{2}}+k_{1}+\frac{v_{d}}{K_{d}+P_{2}}\right)+k_{2}P_{N},$$

$$P_{N}^{\prime }=k_{1}P_{2}-k_{2}P_{N},$$

$$y_{1}=P_{N},$$

**Fig 2:**
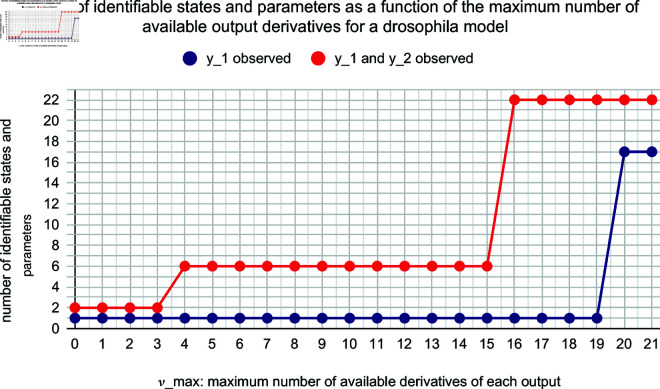
Variation of the number of identifiable parameters with maximum number of available output derivatives in a model of Drosophila period protein. The horizontal axis shows *k*: $\forall i\;(j\leq \nu_{max}↔\;y_{i}^{\left(j\right)}\text{ can be reliably estimated})$. The vertical axis shows the number of identifiable states and parameters in the model. Blue circles represent the case with only one output: $y_{1}=P_{N}$. Red circles represent the case with two outputs: $y_{1}=P_{N}$, *y*_2_ = *M*.

*A local structural identifiability analysis done in [29, p. 737] shows that all states and parameters other than M, $v_{s}$, $v_{m}$, K_m_, and k_s_ are identifiable. However, Algorithm 4 shows that only P_N_ is identifiable using 19 or fewer derivatives of y_1_. If in addition we observe M, it was shown in [29] that all states and parameters are identifiable. However Algorithm 4 shows this is the case only if at least 16 derivatives of the outputs are available. Fig 2 shows the number of states and parameters that are identifiable as a function of the maximum number of derivatives of each output that are available; for example $\nu_{max}=3$ means that exactly $y_{i},y_{i}^{\prime },y_{i}^{\prime \prime },y_{i}^{\left(3\right)}$ are available for all i.*

*Blue circles represent output set $\left\{y_{1}=P_{N}\right\}$ and red circles represent output set $\left\{y_{1}=P_{N},y_{2}=\;M\right\}$.*

**Example 2.8** (NF-κB). *We consider a model of NF-κB regulatory module, as presented below. This model was first introduced in [18], and an identifiability analysis was done in [17]. The model has 15 states, 28 parameters, 4 outputs, and 0 inputs.*


$$x_{1}^{\prime }=-k_{1}x_{1}x_{2}+\frac{1}{333}(-k_{14}x_{1}+k_{15}x_{9}),\;\;\;x_{12}^{\prime }=k_{16}x_{11}+k_{19}x_{10}x_{11}-k_{12}x_{12},$$

$$x_{2}^{\prime }=-k_{1}x_{1}x_{2}+\frac{1}{333}k_{13}x_{8},\;\;\;x_{13}^{\prime }=k_{25}x_{9}x_{11}-k_{17}x_{13},$$

$$x_{3}^{\prime }=k_{1}x_{1}x_{2}-\frac{1}{333}k_{11}x_{3},\;\;\;x_{14}^{\prime }=k_{20}-k_{12}x_{14}-k_{22}x_{14},$$

$$x_{4}^{\prime }=k_{3}+k_{2}x_{2}-k_{4}x_{4},\;\;\;x_{15}^{\prime }=k_{28}x_{7}x_{11}-k_{26}x_{15},$$

$$x_{5}^{\prime }=k_{6}+k_{5}x_{2}-k_{7}x_{5},\;\;\;y_{1}=x_{4},$$

$$x_{6}^{\prime }=k_{9}+k_{8}x_{2}-k_{10}x_{6},\;\;\;y_{2}=x_{5},$$

$$x_{7}^{\prime }=\frac{10}{16667}k_{11}x_{3}-k_{21}x_{7}+k_{1}x_{8}x_{9}-k_{28}x_{7}x_{11},\;\;\;y_{3}=x_{6},$$

$$x_{8}^{\prime }=k_{21}x_{7}-\frac{10}{16667}k_{13}x_{8}-k_{1}x_{8}x_{9}+k_{26}x_{15},\;\;\;y_{4}=x_{10}$$

$$x_{9}^{\prime }=k_{18}x_{5}-k_{23}x_{9}-k_{1}x_{8}x_{9}+\frac{10}{16667}(k_{14}x_{1}-k_{15}x_{9})-k_{25}x_{9}x_{11},$$

$$x_{10}^{\prime }=k_{27}x_{4}-k_{24}x_{10},$$

$$x_{11}^{\prime }=-k_{12}x_{11}-k_{16}x_{11}-k_{28}x_{7}x_{11}-k_{25}x_{9}x_{11}-k_{19}x_{10}x_{11}+k_{17}x_{13}+k_{22}x_{14}+k_{26}x_{15}$$

**Fig 3:**
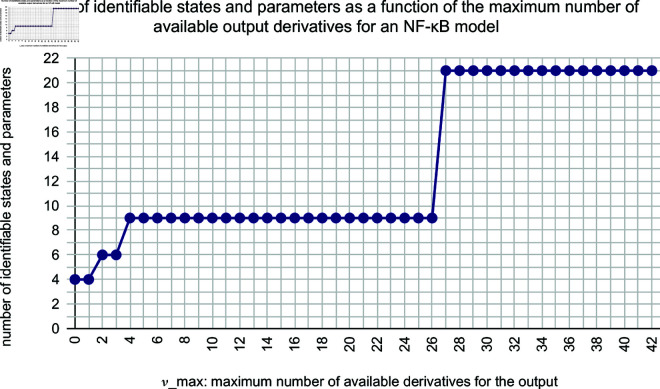
Variation of the number of identifiable parameters with maximum number of available output derivatives in a model of NF-κB regulatory module. The horizontal axis shows $\nu_{max}$: $\forall i\;(j\leq \nu_{max}\;↔\;y_{i}^{\left(j\right)}\text{ can be reliably estimated})$. The vertical axis shows the number of identifiable states and parameters in the model.

*The analysis in [17] shows that 21 states and parameters are identifiable, assuming that all derivatives of the four outputs can be estimated. However, using Algorithm 4, we found that if no more than 26 derivatives are available, then only 9 states and parameters are identifiable. Further analysis is shown in* Fig 3.

### 2.6 Limitations and questions for further research

The definitions and algorithms presented here naturally propose paths for continued research. Our approach requires the modeler to have already determined or assumed the maximum derivatives that can be estimated reliably. This raises the following questions:

1. Given a situation where the noise level is fixed, how can we determine the orders of the highest derivatives that can be reliably used to identify parameter values?
2. Conversely, if one has decided what parameters and states must be identifiable for an experiment to be useful, how can one determine the maximum acceptable noise level so that the derivatives necessary for identification are known precisely enough?

## 3 Theory

The definition of ν-identifiability and the justification of our results are made rigorous in Sect 5. We give a brief summary of this theory here.

The notion of identifiability involves many subtleties. Our precise definition is a type of generic local single-experiment identifiability. This is done in terms of uniqueness (up to a finite set) of parameter and state values given known values of the output derivatives ${𝐲}^{\left(\leq \nu \right)}$ at a certain point in time. We show that if all derivatives of all outputs are available, then our definition is equivalent to the notion of local identifiability given in [15, Def. 2.5].

Our definition of ν-identifiability is intended to capture the property that a modeler really wants to ascertain, but it is often difficult to verify directly. Therefore we show that this property is equivalent to a property involving ranks of matrices, which is straightforward to check using existing software. This is in fact the property alluded to in Algorithm 1.

Although this rank property is straightforward to check, it involves computing ranks of symbolic matrices, which can cost the modeler time. Therefore, to alleviate this problem, we present probabilistic algorithms that substitute random integers for the variables and compute modulo a random prime (Algorithms 3 and 5.3.1). These are modifications of the method described in [29]. We present detailed probabilistic statements that a reader can use to create an implementation without investigating the proofs. We present detailed proofs that the reader can check without excessive pencil and paper computations.

Moreover, our work addresses an oversight in [29] surrounding the probability that a rational expression vanishes when evaluated at a random tuple of integers modulo a random prime. The author assumes that the denominator does not vanish, but then gives a bound for the probability that the numerator does not vanish regardless of this condition. We give a bound on the probability that the numerator does not vanish given that the denominator does not vanish.

## 4 Conclusion

Practical identifiability analysis lies at the heart of model-based data analysis. State-of-the-art numerical methods, such as profile likelihood and MCMC, suffer from the limitation that they require an exhaustive coverage of the parameter space, which is not possible for large and realistic models. This coverage is not needed for structural methods, but these methods traditionally assume that one can determine all derivatives of the measurements *y*. Such derivatives are not available in practice, but the consequences of this unfulfilled assumption have not been examined, because corresponding methods and algorithms are missing. Herein, we present a new definition, ν-identifability, which restricts the analysis to those derivatives of *y* that are available. We also present new algorithms that can determine such identifiability in practice. Applications to previously published models demonstrate that we can determine not only identifiability of parameters, but observability of any model property (Examples 2.5 and 2.6). These results allow us to strengthen previous conclusions. For instance, the previous rejection of a specific model for insulin signaling was based on the model-guided experiments measuring the amount of internalized and dephosphorylated insulin receptors (Example 2.5), and we now show that this model property indeed can be identifiable, using a methodology that does not require coverage of the entire parameter space. Our analysis also shows that the number of parameters identifiable according to traditional structural methods are widely overestimated. If one, e.g., assumes that only up to third order derivatives, instead of all derivatives, are available, the number of identifiable parameters drops from 17 to 1 for the Drosophila model (Fig 2), and from 21 to 4 for an NF-κB model (Fig 3). In both models, the previously obtained identifiability is present only if at least 20 derivatives of all measurement signals are available. Our results bring us one step closer to a structural approach for practical identifiability analysis.

## 5 Appendix: Technical details

### 5.1 Notation and definitions

The notion of identifiability involves many subtleties. This subsection provides the setup necessary for rigorous treatment.

We repeat the setup described previously. Let ℓ be a non-negative integer and let *n*, *m*, and *r* be positive integers. Let $\theta =(\theta_{1},\ldots ,\theta_{\ell })$, $𝐱=(x_{1},\ldots ,x_{n})$, $𝐲=(y_{1},\ldots ,y_{m})$, and $𝐮=(u_{1},\ldots ,u_{r})$. Let $𝐟=(f_{1},\ldots ,f_{n})$ and $𝐠=(g_{1},\ldots ,g_{m})$ be tuples of rational functions in **x**, **u**, and θ over ℂ. This setup determines a class of systems of ODEs with initial conditions, or a model class:

$$\Sigma (t_{0},{𝐱}^{*},\theta ,𝐮)=\left\{ \begin{array}{c} \frac{d𝐱}{dt}=𝐟(𝐱,\theta ,𝐮),\\ 𝐲=𝐠(𝐱,\theta ,𝐮),\\ 𝐱(t_{0})={𝐱}^{*}. \end{array}\right.$$ (2)

Fix non-negative integer ℓ, positive integers *n*, *m*, and *r*, *n*-tuple **f** and *m*-tuple **g** of elements in ℂ(𝐱,θ,𝐮) for the rest of this section. The symbol ℕ denotes the set of non-negative integers.

**Notation 5.1.** *(Differential Algebraic Setting)*

*(a) A differential ring (S,′) is a ring together with a derivation, that is, a function ′:S→S satisfying $\forall a,b\in S\;(a+b{)}^{\prime }=a^{\prime }+b^{\prime }$ and $(ab{)}^{\prime }=a^{\prime }b+ab^{\prime }$. All rings are assumed to be commutative and with multiplicative identity. For a∈S and k∈ℕ, ${\text{a}}^{\left(\text{k}\right)}$ denotes the image of a after k applications of ′. The set $\left\{a^{\left(k\right)}\;|\;k\geq 0\right\}$ will be called the set of derivatives of a.*
*(b) Let (S,′) be a differential ring and let $Z=\{Z_{1},\ldots ,Z_{k}\}$ be a set of indeterminates. Then $S\{Z\}=S\{Z_{1},\ldots ,Z_{k}\}$ denotes the polynomial ring in infinitely many indeterminates $S[Z_{1},Z_{1}^{\prime },Z_{1}^{\prime \prime },\ldots ,Z_{k},Z_{k}^{\prime },Z_{k}^{\prime \prime },\ldots ]$ with derivation ′ extended by $(Z_{i}^{\left(j\right)}{)}^{\prime }=Z_{i}^{\left(j+1\right)}$.*
*(c) Let $\left(S_{1},\;\prime \;\right)$ be a differential subring of $\left(S_{2},\;\prime \;\right)$ and let $Z=\{Z_{1},\ldots ,Z_{k}\}\subseteq S_{2}$. Then $S_{1}\{Z\}=S_{1}\{Z_{1},\ldots ,Z_{k}\}$ denotes the smallest subring of S_2_ containing S_1_ and the derivatives of the elements of Z.*
*(d) Let $\left(S_{1},\;\prime \;\right)$ be a differential ring that is a subring of a differential integral domain $\left(S_{2},\;\prime \;\right)$ and let $Z=\{Z_{1},\ldots ,Z_{k}\}\subseteq S_{2}$. Then $S_{1}\langle Z\rangle =S_{1}\langle Z_{1},\ldots ,Z_{k}\rangle$ denotes the fraction field of S_1_{Z}. This is a differential ring under the extension of ′ via the quotient rule $(a/b{)}^{\prime }=(ba^{\prime }-ab^{\prime })/b^{2}$.*
*(e) For any differential ring containing any elements of ℂ(θ) we assume ′ maps each such element to 0.*
*(f) Let (S,′) be a differential ring. A differential ideal I is an ideal satisfying $\forall a\in I\;a^{\prime }\in I$. For subset A⊆S, we denote by [A] the smallest differential ideal containing A.*
*(g) For an ideal I and element a in a ring S, we denote $I:a^{\infty }=\{s\in S|\exists k:a^{k}s\in I\}$. This set is also an ideal in S.*
*(h) Write 𝐟 and 𝐠 as fractions of elements of ℂ[𝐱,θ,𝐮], let Q∈ℂ[𝐱,θ,𝐮] be the LCM of the denominators, and let 𝐅 and 𝐆 be such that 𝐟=𝐅/Q and 𝐠=𝐆/Q. Note that Q is unique up to multiplication by an element of ℂ and choice of Q will not affect our definitions and results. We define the differential ideal of Σ as $𝒥=[Q{𝐱}^{\prime }-𝐅,Q𝐲-𝐆]:Q^{\infty }\subseteq \mathbb{C}[\theta ]\{𝐱,𝐲,𝐮\}$. This is a prime (differential) ideal (cf [15, Lemma 3.2]) and thus R:=ℂ[θ]{𝐮,𝐱,𝐲}/𝒥 is a differential ring that is an integral domain.*
*(i) For $\nu \in {\mathbb{N}}^{m}$, we denote by ${𝐲}^{\left(\leq \nu \right)}$ the subset $\left\{y_{i}^{\left(j_{i}\right)}\;|\;1\leq i\leq m,0\leq j_{i}\leq \nu_{i}\right\}$ of R. We define $\nu_{max}:=\max(\nu_{1},\ldots ,\nu_{m})$.*

**Notation 5.2.1** *(Analytic Setting)*

*(a) Let $t_{0}\in \mathbb{C}$. Then ${\mathbb{C}}^{\infty }(t_{0})$ denotes the set of all functions that are complex analytic in some neighborhood of t = t_0_.*
*(b) Let $t_{0}\in \mathbb{C}$. Then $\Omega^{t_{0}}\subseteq {\mathbb{C}}^{n}\times {\mathbb{C}}^{\ell }\times ({\mathbb{C}}^{\infty }(t_{0}){)}^{r}$ denotes the complement to the set where at least one of the denominators of **f** or **g** vanishes at t = t_0_.*
*(c) For $t_{0}\in \mathbb{C}$ and h∈ℂ(𝐱,θ), let $\Omega_{h}^{t_{0}}=\{({\hat{𝐱}}^{*},\hat{\theta },\hat{𝐮})\in \Omega^{t_{0}}\;|\;h({\hat{𝐱}}^{*},\hat{\theta })\text{ is defined}\}$.*
*(d) For $t_{0}\in \mathbb{C}$ and $({\hat{𝐱}}^{*},\hat{\theta },\hat{𝐮})\in \Omega^{t_{0}}$, let $X(t_{0},{\hat{𝐱}}^{*},\hat{\theta },\hat{𝐮})\in ({\mathbb{C}}^{\infty }(t_{0}){)}^{n}$ and $Y(t_{0},{\hat{𝐱}}^{*},\hat{\theta },\hat{𝐮})\in ({\mathbb{C}}^{\infty }(t_{0}){)}^{m}$ denote the unique solution of the initial value problem $\Sigma (t_{0},{\hat{𝐱}}^{*},\hat{\theta },\hat{𝐮})$ (cf. [1, Theorem 2.2.2]). For appropriate integers j and k, let $Y_{j}^{\left(k\right)}(t_{0},{\hat{𝐱}}^{*},\hat{\theta },\hat{𝐮})$ denote the k-th derivative of the j-th component of $Y(t_{0},{\hat{𝐱}}^{*},\hat{\theta },\hat{𝐮})$.*
*(e) For s∈ℕ, a subset $U\subseteq {\mathbb{C}}^{s}$ is called Zariski open if there exists P in $\mathbb{C}[Z_{1},\ldots ,Z_{s}]$ such that U is the complement to the zero set of P.*
*(f) For $s\in {\mathbb{N}}^{+}$ and $t_{0}\in \mathbb{C}$, a subset $U\subseteq ({\mathbb{C}}^{\infty }(t_{0}){)}^{s}$ is called Zariski open if there exists P in $\mathbb{C}\{Z_{1},\ldots ,Z_{s}\}$ such that*
$$U=\{\hat{z}\in ({\mathbb{C}}^{\infty }(t_{0}){)}^{s}|P(\hat{z}){|}_{t=t_{0}}\neq 0\}.$$
*(g) For s∈ℕ, $t_{0}\in \mathbb{C}$, and $W={\mathbb{C}}^{s}$ or $({\mathbb{C}}^{\infty }(t_{0}){)}^{s}$, the set of all nonempty Zariski open subsets of W will be denoted by τ(W).*
*(h) A subset V⊆ℂ is called codiscrete if for any distinct $c_{1},c_{2}\in \mathbb{C}\backslash V$ there exist disjoint open sets $W_{1}∋c_{1}$ and $W_{2}∋c_{2}$ such that $W_{1}\cap (\mathbb{C}\backslash V)=\{c_{1}\}$ and $W_{2}\cap (\mathbb{C}\backslash V)=\{c_{2}\}$.*

**Notation 5.3.** *Let h∈ℂ(𝐱,θ)⟨𝐮⟩, let $t_{1}\in \mathbb{C}$, and let $\hat{p}\in \Omega_{h}^{t_{1}}$.*

*(a) Substituting $\left(X(t_{1},\hat{p}),\hat{\theta },\hat{𝐮}\right)$ for (𝐱,θ,𝐮) in h gives a function of one variable. The image of h under this substitution will be denoted by $h(t_{1},\hat{p})(t)$.*
*(b) The connected component containing t_1_ of the intersection of the domains of $h(t_{1},\hat{p})(t)$, $\hat{𝐮}(t)$, $X(t_{1},\hat{p})(t)$ and $Y(t_{1},\hat{p})(t)$ will be denoted by $D_{h}(t_{1},\hat{p})$.*

**Definition 5.4.** *Let $\nu \in {\mathbb{N}}^{m}$. Let h∈ℂ(𝐱,θ). The expression h is said to be ν-identifiable if*


$$\exists \Psi \in \tau ({\mathbb{C}}^{n+\ell })\;\exists U\in \tau (({\mathbb{C}}^{\infty }(0){)}^{r})\;\forall \hat{p}:=({\hat{𝐱}}^{*},\hat{\theta },\hat{𝐮})\in \Omega_{h}^{0}\cap (\Psi \times U)$$

$$\exists \text{ codiscrete }V\subseteq \mathbb{C}\;\forall t_{0}\in V\cap D_{h}(0,\hat{p})\;|S_{h}(t_{0},{\hat{𝐱}}^{*},\hat{\theta },\hat{𝐮})|\text{ is finite,}$$

*where*


$$S_{h}(t_{0},{\hat{𝐱}}^{*},\hat{\theta },\hat{𝐮})=\{h({\tilde{𝐱}}^{*},\tilde{\theta })\;|\;\tilde{p}:=({\tilde{𝐱}}^{*},\tilde{\theta },\hat{𝐮})\in \Omega_{h}^{t_{0}}\text{ and }\;Y^{\left(\leq \nu \right)}(t_{0},\tilde{p})(t_{0})=\;Y^{\left(\leq \nu \right)}(0,\hat{p})(t_{0})\}.$$

One can interpret this definition as follows: If at some time *t*_0_, not belonging to a certain discrete “singular” set, we know the exact values of $Y^{\left(\leq \nu \right)}(t_{0})$, then if we consider all possible values of the state variables and parameters at *t*_0_ that produce these values of $Y_{i}^{\left(j\right)}(t_{0})$, and then we consider the set of values of *h* obtained by evaluating *h* at these values, this set is finite.

#### 5.1.1 Examples to illustrate the definition.

**Example 5.5.** *Consider the system*


$$\Sigma :\;x_{1}^{\prime }=\theta_{1}\theta_{2}x_{1},\;y_{1}=x_{1},\;y_{2}=\theta_{2},\;x_{1}(t_{0})=x_{1}^{*}.$$

*We show that $\theta_{1}$ is (1,0)-identifiable. Note that n = 1 and m = 2. Since two parameters occur we can take ℓ to be any integer at least 2. Although no inputs occur the definitions require that we take r to be at least 1. Set ℓ=2 and r = 1. Let $\Psi =\{({\hat{x}}_{1}^{*},{\hat{\theta }}_{1},{\hat{\theta }}_{2})\in {\mathbb{C}}^{3}\;|\;{\hat{\theta }}_{2}{\hat{x}}_{1}^{*}\neq 0\}$ and let $U={\mathbb{C}}^{\infty }(0)$. Note that since there are no denominators we have $\Omega_{\theta_{1}}^{t_{0}}={\mathbb{C}}^{3}\times {\mathbb{C}}^{\infty }(t_{0})$ for all t_0_. Fix $\hat{p}:=({\hat{x}}_{1}^{*},{\hat{\theta }}_{1},{\hat{\theta }}_{2},\hat{u})\in \Omega_{\theta_{1}}^{0}\cap (\Psi \times U)$. Let V=ℂ and fix $t_{0}\in V\cap D_{\theta_{1}}(0,\hat{p})$. It is trivial to obtain a formula for the solution of this system, and we have*


$$Y_{1}(0,\hat{p})(t)={\hat{x}}_{1}^{*}e^{{\hat{\theta }}_{1}{\hat{\theta }}_{2}t},\;Y_{2}(0,\hat{p})(t)={\hat{\theta }}_{2}.$$

*Let $\tilde{p}:=({\tilde{x}}_{1}^{*},{\tilde{\theta }}_{1},{\tilde{\theta }}_{2},\hat{u})\in \Omega_{\theta_{1}}^{t_{0}}$, and note that*

$$Y_{1}(t_{0},\tilde{p})(t)={\tilde{x}}_{1}^{*}e^{{\tilde{\theta }}_{1}{\tilde{\theta }}_{2}(t-t_{0})},\;Y_{2}(t_{0},\tilde{p})(t)={\tilde{\theta }}_{2}.$$ (3)

*For ν=(1,0), the condition $Y^{\left(\leq \nu \right)}(t_{0},\tilde{p})(t_{0})=Y^{\left(\leq \nu \right)}(0,\hat{p})(t_{0})$ gives*

$${\hat{x}}_{1}^{*}e^{{\hat{\theta }}_{1}{\hat{\theta }}_{2}t_{0}}={\tilde{x}}_{1}^{*},\;{\hat{x}}_{1}^{*}{\hat{\theta }}_{1}{\hat{\theta }}_{2}e^{{\hat{\theta }}_{1}{\hat{\theta }}_{2}t_{0}}={\tilde{x}}_{1}^{*}{\tilde{\theta }}_{1}{\tilde{\theta }}_{2},\;{\hat{\theta }}_{2}={\tilde{\theta }}_{2}.$$ (4)

*By the definition of Ψ, it follows that no side of the first or third of these equations is 0. Dividing the second equation by the product of the first and third proves that ${\hat{\theta }}_{1}={\tilde{\theta }}_{1}$. Since $\theta_{1}(\tilde{p})={\tilde{\theta }}_{1}$, we see that $S_{\theta_{1}}(t_{0},\hat{p})=\{{\hat{\theta }}_{1}\}$ and thus has cardinality 1. We conclude that $\theta_{1}$ is (1,0)-identifiable.*

*We now show that $\theta_{1}$ is not (0,0)-identifiable. Fix $\Psi \in \tau ({\mathbb{C}}^{3})$ and $U\in \tau ({\mathbb{C}}^{\infty }(0))$. Let $\hat{p}:=({\hat{x}}_{1}^{*},{\hat{\theta }}_{1},{\hat{\theta }}_{2},\hat{u})\in \Omega_{\theta_{1}}^{0}\cap (\Psi \times U)$. Fix codiscrete V, and let $t_{0}\in V\cap D_{\theta_{1}}(0,\hat{p})$. Using the first and third equations of* (4), *we see that for all k∈ℂ the tuple ${\tilde{p}}_{k}:=({\hat{x}}_{1}^{*}e^{{\hat{\theta }}_{1}{\hat{\theta }}_{2}t_{0}},k,{\hat{\theta }}_{2},\hat{u})$ satisfies the condition $Y^{\left(\leq \nu \right)}(t_{0},{\tilde{p}}_{k})(t_{0})=Y^{\left(\leq \nu \right)}(0,\hat{p})(t_{0})$ for ν=(0,0). Therefore*


$$S_{\theta_{1}}(t_{0},\hat{p})⊇\{k\;|\;k\in \mathbb{C}\},$$

*and the right-hand side is infinite. We conclude that $\theta_{1}$ is not* (0,0)-*identifiable.*

The next example includes an input and demonstrates why sometimes a discrete set must be excluded from *V*.

**Example 5.6.** *Consider the system*


$$\Sigma :\;x_{1}^{\prime }=\frac{1}{x_{1}},\;x_{2}^{\prime }=f_{2}(x_{1},x_{2},u_{1})$$

$$y_{1}=x_{2}(x_{1}-u_{1}),\;y_{2}=x_{1}-u_{1},\;x_{1}(t_{0})=x_{1}^{*},\;x_{2}(t_{0})=x_{2}^{*},$$

*where f_2_ is some rational function.*

*We have n = 2 and m = 2. Take ℓ=0 and r = 1. We show that x_2_ is (0,0)-identifiable. We have $\Omega_{x_{2}}^{0}=\{({\hat{x}}_{1}^{*},{\hat{x}}_{2}^{*},{\hat{u}}_{1})\;|\;{\hat{x}}_{1}^{*}\cdot (\text{denom}f_{2})({\hat{x}}_{1}^{*},{\hat{x}}_{2}^{*},{\hat{u}}_{1})\neq 0\}$. Let $\Psi ={\mathbb{C}}^{2}$ and let $U=\{z\in {\mathbb{C}}^{\infty }(0)\;|\;z(0)z^{\prime }(0)-1\neq 0\}$. Let $\hat{p}:=({\hat{x}}_{1}^{*},{\hat{x}}_{2}^{*},{\hat{u}}_{1})\in \Omega_{x_{2}}^{0}\cap (\Psi \times U)$. Note that because of the way U was defined, $X_{1}(0,\hat{p})-{\hat{u}}_{1}$ is not the zero function. Let V be the complement of the vanishing of $X_{1}(0,\hat{p})-{\hat{u}}_{1}$, which is necessarily codiscrete since $X_{1}(0,\hat{p})$ and ${\hat{u}}_{1}$ are analytic. Let $t_{0}\in V\cap D_{x_{2}}(0,\hat{p})$. If $\tilde{p}=({\tilde{x}}_{1}^{*},{\tilde{x}}_{2}^{*},{\hat{u}}_{1})$ is such that $Y_{i}(t_{0},\tilde{p})(t_{0})=Y_{i}(0,\hat{p})(t_{0})$ for i=1,2 then it follows that*


$$x_{2}(\tilde{p})={\tilde{x}}_{2}^{*}=X_{2}(t_{0},\tilde{p})(t_{0})=\frac{Y_{1}(t_{0},\tilde{p})(t_{0})}{Y_{2}(t_{0},\tilde{p})(t_{0})}=\frac{Y_{1}(0,\hat{p})(t_{0})}{Y_{2}(0,\hat{p})(t_{0})}=X_{2}(0,\hat{p})(t_{0}).$$

*Since $t_{0}\in D_{x_{2}}(0,\hat{p})$ we know that $Y_{1}(0,\hat{p})(t_{0})$ and $Y_{2}(0,\hat{p})(t_{0})$ are defined and since $t_{0}\in V$ we know that $Y_{2}(0,\hat{p})(t_{0})\neq 0$. Thus $|S_{x_{2}}(t_{0},\hat{p})|=1$ and we conclude that x_2_ is (0,0)-identifiable.*

#### 5.1.2 ν-identifiable implies single-experiment locally identifiable and the converse is true for sufficiently large ν.

Many notions of identifiability are used in the literature. Some of these are stated and compared in [5]. In general, Definition 5.4 is not equivalent to any published definition as far as we are aware.

Definition 5.4 can be viewed as a type of single-experiment generic local identifiability. It is generic because there is a (possibly empty) set of numerical values of parameters and input functions for which *h* cannot be recovered but if the true values of the parameters and inputs lie outside this set *h* can be recovered. It is local because we allow *h* to lie in a finite set. One could change “is finite” to “equals one” to create a definition of a globally ν-identifiable function, however we do not address this in the current work. It is “single-experiment” because it refers to only one instance of the model. This is in contrast to a multi-experiment approach where one observes multiple instances of the model, usually with the same equation parameters but different initial conditions.

Since the equations of Σ are rational in **x**, θ, and **u**, there exist relations among sufficiently high derivatives of **y** and hence it is not meaningful to consider $\nu_{i}$ beyond a certain order. This is made precise by the following proposition.

**Proposition 5.7.** *Let h∈ℂ(𝐱,θ) and $\nu \in {\mathbb{N}}^{m}$. For each i=1,…,m let $\mu_{i}$ be the greatest non-negative integer such that the set $y_{i},y_{i}^{\prime },\ldots ,y_{i}^{\left(\mu_{i}\right)}$ is not algebraic over ℂ⟨𝐮⟩. Then h is ν-identifiable if and only if h is $\left(\min(\nu_{1},\mu_{1}),\ldots ,\min(\nu_{m},\mu_{m})\right)$-identifiable. Moreover, $\forall i\;\mu_{i}\leq n+\ell -1$.*

*Proof*: Since the transcendence degree of ℂ⟨𝐮⟩(𝐱,θ) over ℂ⟨𝐮⟩ is n+ℓ, it must be that $y_{i},y_{i}^{\prime },\ldots ,y_{i}^{\left(n+\ell \right)}$ is algebraic over ℂ⟨𝐮⟩. Hence such a $\mu_{i}$ exists and moreover $\mu_{i}\leq n+\ell -1$.

For the ⇐ direction, note that if $𝐚,𝐛\in {\mathbb{N}}^{m}$ are such that $a_{i}\geq b_{i}$ for all *i*, it follows from the definition that *h* is **b**-identifiable implies *h* is **a**-identifiable.

We now address the ⇒ direction. Suppose *h* is ν-identifiable. By Proposition 5.12, *h* is algebraic over $\mathbb{C}\langle 𝐮\rangle ({𝐲}^{\left(\leq \nu \right)})$. For any *i*, by writing an algebraic dependence of $y_{i},y_{i}^{\prime },\ldots ,y_{i}^{\left(\mu_{i}+1\right)}$ over ℂ⟨𝐮⟩ and differentiating (noting that the field has characteristic 0), we see that for all $s>\mu_{i}$ the element $y_{i}^{\left(s\right)}$ is algebraic over $\mathbb{C}\langle 𝐮\rangle (y_{i},\ldots ,y_{i}^{\left(\mu_{i}\right)})$. It follows that *h* is algebraic over $\mathbb{C}\langle 𝐮\rangle (\{y_{i},\ldots ,y_{i}^{\left(\min(\nu_{i},\mu_{i})\right)}{\}}_{i=1,\ldots ,m})$. By Proposition 5.12 *h* is $\left(\min(\nu_{1},\mu_{1}),\ldots ,\min(\nu_{m},\mu_{m})\right)$-identifiable. ◻

**Definition 5.8.** *Let h∈ℂ(𝐱,θ). The expression h is said to be single-experiment locally identifiable (SELI) if*


$$\exists \Psi \in \tau ({\mathbb{C}}^{n+\ell })\;\exists U\in \tau (({\mathbb{C}}^{\infty }(0){)}^{r})\;\forall \hat{p}:=({\hat{𝐱}}^{*},\hat{\theta },\hat{𝐮})\in \Omega_{h}^{0}\cap (\Psi \times U)\;|S_{h}({\hat{𝐱}}^{*},\hat{\theta },\hat{𝐮})|\text{ is finite,}$$

*where*


$$S_{h}({\hat{𝐱}}^{*},\hat{\theta },\hat{𝐮})=\left\{h({\tilde{𝐱}}^{*},\tilde{\theta })\;|\;\tilde{p}:=({\tilde{𝐱}}^{*},\tilde{\theta },\hat{𝐮})\in \Omega_{h}^{0}\text{ and }Y(0,\tilde{p})=\;Y(0,\hat{p})\right\}.$$

For h∈𝐱∪θ, Definition 5.8 is equivalent to the definition of local identifiability given in Definition 2.5 of [15] (generalization to multiple inputs is asserted in Remark 2.2). In [15, Prop. 3.4 (a) ⇔ (c)], it was shown that for h∈𝐱∪θ, *h* is SELI if and only if *h* is algebraic over ℂ⟨𝐲,𝐮⟩. We extend this result to arbitrary h∈ℂ(𝐱,θ).

**Proposition 5.9.** *Let h∈ℂ(𝐱,θ). Then h is SELI if and only if h is algebraic over ℂ⟨𝐲,𝐮⟩.*

*Proof*: Let $\Sigma_{1}(0,{𝐱}^{*},x_{n+1}^{*},\theta ,𝐮)$ be the system obtained by adding to $\Sigma (0,{𝐱}^{*},\theta ,𝐮)$ the equations $x_{n+1}^{\prime }=\sum_{i=1}^{n}f_{i}\frac{\partial h}{\partial x_{i}}$, $y_{m+1}=x_{n+1}-h$, and $x_{n+1}(0)=x_{n+1}^{*}$. Note that $\Omega_{h}^{0}$ in Σ is the projection of $\Omega_{x_{n+1}}^{0}$ onto all coordinates but *x*_*n* + 1_. We divide the proof into the following three steps: *h* is Σ-SELI $\overset{1}{\Leftrightarrow }x_{n+1}$ is $\Sigma_{1}$-SELI $\overset{2}{\Leftrightarrow }x_{n+1}$ is algebraic over $\mathbb{C}\langle 𝐲,y_{m+1},𝐮\rangle \overset{3}{\Leftrightarrow }$ *h* is algebraic over ℂ⟨𝐲,𝐮⟩.

We show *h* is Σ-SELI $⟺x_{n+1}$ is $\Sigma_{1}$-SELI. Suppose *h* is Σ-SELI. Let Ψ and *U* be as required by the definition. Define $\Psi_{1}=\Psi \times \mathbb{C}$. Let $\left({\hat{𝐱}}^{*},{\hat{x}}_{n+1}^{*},\hat{\theta },\hat{𝐮})\in \Omega_{x_{n+1}}^{0}\cap (\Psi_{1}\times U\right)$. We verify that $\left|S_{x_{n+1}}({\hat{𝐱}}^{*},{\hat{x}}_{n+1}^{*},\hat{\theta },\hat{𝐮})|=|S_{h}({\hat{𝐱}}^{*},\hat{\theta },\hat{𝐮})\right|$. Since $Y_{m+1}^{\prime }=0$, we have


$$S_{x_{n+1}}({\hat{𝐱}}^{*},{\hat{x}}_{n+1}^{*},\hat{\theta },\hat{𝐮})=\{{\tilde{x}}_{n+1}^{*}\;|\;({\tilde{𝐱}}^{*},{\tilde{x}}_{n+1}^{*},\tilde{\theta },\hat{𝐮})\in \Omega_{x_{n+1}}^{0}\;\text{ and }Y(0,({\tilde{𝐱}}^{*},{\tilde{x}}_{n+1}^{*},\tilde{\theta },\hat{𝐮}))=\;Y(0,({\hat{𝐱}}^{*},{\hat{x}}_{n+1}^{*},\hat{\theta },\hat{𝐮}))\;\text{ and }{\tilde{x}}_{n+1}^{*}-h({\tilde{𝐱}}^{*},\tilde{\theta })={\hat{x}}_{n+1}^{*}-h({\hat{𝐱}}^{*},\hat{\theta })\},$$

where $Y=(Y_{1},\ldots ,Y_{m})$. It follows that


$$S_{x_{n+1}}({\hat{𝐱}}^{*},{\hat{x}}_{n+1}^{*},\hat{\theta },\hat{𝐮})\;=\{h({\tilde{𝐱}}^{*},\tilde{\theta })+{\hat{x}}_{n+1}^{*}-h({\hat{𝐱}}^{*},\hat{\theta })\;|\;({\tilde{𝐱}}^{*},{\tilde{x}}_{n+1}^{*},\tilde{\theta },\hat{𝐮})\in \Omega_{x_{n+1}}^{0}\text{ and }\;\;\;\;\;Y(0,({\tilde{𝐱}}^{*},{\tilde{x}}_{n+1}^{*},\tilde{\theta },\hat{𝐮}))=\;Y(0,({\hat{𝐱}}^{*},{\hat{x}}_{n+1}^{*},\hat{\theta },\hat{𝐮}))\}\;=\{h({\tilde{𝐱}}^{*},\tilde{\theta })+{\hat{x}}_{n+1}^{*}-h({\hat{𝐱}}^{*},\hat{\theta })\;|\;({\tilde{𝐱}}^{*},\tilde{\theta },\hat{𝐮})\in \Omega_{h}^{0}\text{ and }\;\;\;\;\;Y(0,({\tilde{𝐱}}^{*},\tilde{\theta },\hat{𝐮}))=\;Y(0,({\hat{𝐱}}^{*},\hat{\theta },\hat{𝐮}))\},$$

where $\Omega_{h}^{0}$ is considered a subset of ${\mathbb{C}}^{n+\ell }\times {\mathbb{C}}^{\infty }(0)$. Thus


$$\left|S_{x_{n+1}}({\hat{𝐱}}^{*},{\hat{x}}_{n+1}^{*},\hat{\theta },\hat{𝐮})\right|=|\{h({\tilde{𝐱}}^{*},\tilde{\theta })\;|\;({\tilde{𝐱}}^{*},\tilde{\theta },\hat{𝐮})\in \Omega_{h}^{0}\text{ and }\;\;\;\;\;Y(0,({\tilde{𝐱}}^{*},\tilde{\theta },\hat{𝐮}))=\;Y(0,({\hat{𝐱}}^{*},\hat{\theta },\hat{𝐮}))\}|\;=\left|S_{h}({\hat{𝐱}}^{*},\hat{\theta },\hat{𝐮})\right|,$$

and we know that $|S_{h}({\hat{𝐱}}^{*},\hat{\theta },\hat{𝐮})|<\infty$ because *h* is Σ-SELI. This completes the first direction. Now suppose *x*_*n* + 1_ is $\Sigma_{1}$-SELI. Let $\Psi_{1}$ and *U* be as required by the definition. Define Ψ to be the projection of $\Psi_{1}$ onto all coordinates other than *x*_*n* + 1_. Let $\left({\hat{𝐱}}^{*},\hat{\theta },\hat{𝐮})\in \Omega_{h}^{0}\cap (\Psi \times U\right)$. Let ${\hat{x}}_{n+1}^{*}$ be such that $\left({\hat{𝐱}}^{*},{\hat{x}}_{n+1}^{*},\hat{\theta },\hat{𝐮})\in \Omega_{x_{n+1}}^{0}\cap (\Psi_{1}\times U\right)$. Such a ${\hat{x}}_{n+1}^{*}$ exists because of the way Ψ was defined. Now


$$S_{h}({\hat{𝐱}}^{*},\hat{\theta },\hat{𝐮})=\{h({\tilde{𝐱}}^{*},\tilde{\theta })\;|\;({\tilde{𝐱}}^{*},\tilde{\theta },\hat{𝐮})\in \Omega_{h}^{0}\text{ and }Y(0,({\tilde{𝐱}}^{*},\tilde{\theta },\hat{𝐮}))=\;Y(0,({\hat{𝐱}}^{*},\hat{\theta },\hat{𝐮}))\}\;=\{h({\tilde{𝐱}}^{*},\tilde{\theta })\;|\;({\tilde{𝐱}}^{*},{\tilde{x}}_{n+1}^{*},\tilde{\theta },\hat{𝐮})\in \Omega_{x_{n+1}}^{0}\;\;\;\;\;\text{ and }Y(0,({\tilde{𝐱}}^{*},{\tilde{x}}_{n+1}^{*},\tilde{\theta },\hat{𝐮}))=\;Y(0,({\hat{𝐱}}^{*},{\tilde{x}}_{n+1}^{*},\hat{\theta },\hat{𝐮}))\;\;\;\;\;\text{ and }{\tilde{x}}_{n+1}^{*}-h({\tilde{𝐱}}^{*},\tilde{\theta })={\hat{x}}_{n+1}^{*}-h({\hat{𝐱}}^{*},\hat{\theta })\}\;=\{{\tilde{x}}_{n+1}^{*}-{\hat{x}}_{n+1}^{*}+h({\hat{𝐱}}^{*},\hat{\theta })\;|\;({\tilde{𝐱}}^{*},{\tilde{x}}_{n+1}^{*},\tilde{\theta },\hat{𝐮})\in \Omega_{x_{n+1}}^{0}\text{ and }\;\;\;\;\;Y_{i}(0,({\tilde{𝐱}}^{*},{\tilde{x}}_{n+1}^{*},\tilde{\theta },\hat{𝐮}))=\;Y_{i}(0,({\hat{𝐱}}^{*},{\hat{x}}_{n+1}^{*},\hat{\theta },\hat{𝐮})),\;\;\;\;\;i=1,\ldots ,m+1\}.$$

Hence


$$|S_{h}({\hat{𝐱}}^{*},\hat{\theta },\hat{𝐮})|=|\{{\tilde{x}}_{n+1}^{*}\;|\;({\tilde{𝐱}}^{*},{\tilde{x}}_{n+1}^{*},\tilde{\theta },\hat{𝐮})\in \Omega_{x_{n+1}}^{0}\text{ and }\;\;\;\;Y_{i}(0,({\tilde{𝐱}}^{*},{\tilde{x}}_{n+1}^{*},\tilde{\theta },\hat{𝐮}))=\;Y_{i}(0,({\hat{𝐱}}^{*},{\tilde{x}}_{n+1}^{*},\hat{\theta },\hat{𝐮})),\;\;\;\;i=1,\ldots ,m+1\}|\;=|S_{x_{n+1}}({\hat{𝐱}}^{*},{\hat{x}}_{n+1}^{*},\hat{\theta },\hat{𝐮})|,$$

and we know that $|S_{x_{n+1}}({\hat{𝐱}}^{*},{\hat{x}}_{n+1}^{*},\hat{\theta },\hat{𝐮})|<\infty$ because *x*_*n* + 1_ is $\Sigma_{1}$-SELI.

From [15, Prop. 3.4 (a) ⇔ (c)], we have that *x*_*n* + 1_ is $\Sigma_{1}$-SELI $⟺x_{n+1}$ is algebraic over $\mathbb{C}\langle 𝐲,y_{m+1},𝐮\rangle$.

We now show that *h* is algebraic over $\mathbb{C}\langle 𝐲,𝐮\rangle ⟺x_{n+1}$ is algebraic over $\mathbb{C}\langle 𝐲,y_{m+1},𝐮\rangle$. Suppose *h* is algebraic over ℂ⟨𝐲,𝐮⟩. Then $x_{n+1}=h+y_{m+1}$ is algebraic over $\mathbb{C}\langle 𝐲,y_{m+1},𝐮\rangle$. We now address the other direction. Let ℱ=ℂ⟨𝐲,𝐮⟩ and let ${ℱ}_{1}=\mathbb{C}\langle 𝐲,y_{m+1},𝐮\rangle$. Suppose *x*_*n* + 1_ is algebraic over ${ℱ}_{1}$. Then $h=x_{n+1}-y_{m+1}$ is algebraic over ${ℱ}_{1}$. Let $\sum_{i=0}^{k}a_{i}Z^{i}$ be the minimal polynomial of *h* over ${ℱ}_{1}$. Suppose *y*_*m* + 1_ appears in some *a*_*i*_. Let α be the differential field automorphism on $\mathbb{C}(𝐱,x_{n+1},\theta )\langle 𝐮\rangle$ such that ℂ(𝐱,θ)⟨𝐮⟩ is fixed pointwise, $\alpha (y_{m+1})=y_{m+1}+1$, and $\alpha (x_{n+1})=x_{n+1}+1$. Now $\sum_{i=0}^{k}(a_{i}-\alpha (a_{i}))Z^{i}$ is a non-zero polynomial of lower degree with coefficients in ${ℱ}_{1}$ that has *h* as a root. Thus we have a contradiction. Therefore all the *a*_*i*_ lie in ℱ and *h* is algebraic over ℱ. ◻

This leads to the main result of this section.

**Corollary 5.10.** *Let h∈ℂ(𝐱,θ) and $\nu \in {\mathbb{N}}^{m}$.*

1. *h is ν-identifiable ⇒ h is SELI.*
2. *If $\forall i\;\nu_{i}\geq n+\ell -1$, then h is SELI ⇒ h is ν-identifiable.*

*Proof*: Suppose *h* is ν-identifiable. Then by Proposition 5.12 we know *h* is algebraic over $\mathbb{C}\langle 𝐮\rangle ({𝐲}^{\left(\leq \nu \right)})$. It follows that *h* is algebraic over ℂ⟨𝐲,𝐮⟩ and then by Proposition 5.9 we have that *h* is SELI.

Suppose that $\forall i\;\nu_{i}\geq n+\ell -1$. Then by the arguments presented in Proposition 5.7 we have $\mathbb{C}\langle 𝐮\rangle ({𝐲}^{\left(\leq \nu \right)})=\mathbb{C}\langle 𝐮,𝐲\rangle$. Suppose *h* is SELI. By Proposition 5.9 we have that *h* is algebraic over ℂ⟨𝐮,𝐲⟩, which equals $\mathbb{C}\langle 𝐮\rangle ({𝐲}^{\left(\leq \nu \right)})$. By Proposition 5.12 we have that *h* is ν-identifiable. ◻

### 5.2 Proof of algorithms

The definition of ν-identifiability is stated in terms of analytic functions. The Proposition 5.12 gives a correspondence between the analytic property and an algebraic property. Its proof will use the following lemma.

**Lemma 5.11.** *Let $t_{1},t_{2}\in \mathbb{C}$, and let $\hat{p}\in \Omega^{t_{1}}$. The map*


$$h\mapsto h(t_{1},\hat{p})(t_{2})$$

*gives a ℂ-algebra homomorphism S→ℂ, where S is the ring $\left\{h\in \text{Frac}(R)\;|\;t_{2}\in D_{h}(t_{1},\hat{p})\right\}$. Moreover, under this map $𝐲\mapsto Y(t_{1},\hat{p})(t_{2})$.*

*Proof*: Denote the stated map by φ. Since $X(t_{1},{\hat{𝐱}}^{*},\hat{\theta },\hat{𝐮}),\hat{\theta },\hat{𝐮}$ satisfy all the relations among 𝐱,θ,𝐮 and no denominator of *S* is sent to 0, we see that φ is a homomorphism on *S*. Now 𝐲=𝐠(𝐱,θ,𝐮), so $\varphi (𝐲)=𝐠(\varphi (𝐱),\varphi (\theta ),\varphi (𝐮))=𝐠(X(t_{1},{\hat{𝐱}}^{*},\hat{\theta },\hat{𝐮}),\hat{\theta },\hat{𝐮})(t_{2})$. The existence and uniqueness theorem guarantees the existence and uniqueness of $X(t_{1},{\hat{𝐱}}^{*},\hat{\theta },\hat{𝐮})$, and $Y(t_{1},{\hat{𝐱}}^{*},\hat{\theta },\hat{𝐮})$ is given by $𝐠(X(t_{1},{\hat{𝐱}}^{*},\hat{\theta },\hat{𝐮}),\hat{\theta },\hat{𝐮})$. ◻

**Proposition 5.12.** *Let $\nu \in {\mathbb{N}}^{m}$. Expression h∈ℂ(𝐱,θ) is ν-identifiable if and only if h is algebraic over the subfield $\mathbb{C}\langle 𝐮\rangle ({𝐲}^{\left(\leq \nu \right)})$ of *Frac*(*R*).*

*Proof*: Part 1: ⇐ Assume that *h* is algebraic over $\mathbb{C}\langle 𝐮\rangle ({𝐲}^{\left(\leq \nu \right)})$. Consider the minimal polynomial of *h* over $\mathbb{C}\langle 𝐮\rangle ({𝐲}^{\left(\leq \nu \right)})$. Clearing denominators, we obtain the polynomial $P(Z):=\sum_{i=0}^{k}a_{i}Z^{i}$, where each $a_{i}\in \mathbb{C}\{𝐮\}[{𝐲}^{\left(\leq \nu \right)}]$ and P(h)=0. By [16, Corollary 6.6], there exist $\Psi \in \tau ({\mathbb{C}}^{n+\ell })$ and $U\in \tau (({\mathbb{C}}^{\infty }(0){)}^{r})$ such that for all $\hat{p}\in (\Psi \times U)\cap \Omega_{h}^{0}$ it holds that $a_{k}(0,\hat{p})(t)$ (see Notation 5.3) is not the zero function. Fix such Ψ and *U* and choose a $\hat{p}\in \Omega_{h}^{0}\cap (\Psi \times U)$. Let *V* be the complement of the vanishing of $a_{k}(0,\hat{p})(t)$. Since $a_{k}(0,\hat{p})(t)$ is analytic about 0, we know that *V* is codiscrete.

Fix $t_{0}\in V\cap D_{h}(0,\hat{p})$. Let ${\tilde{𝐱}}^{*}$, $\tilde{\theta }$ be such that $\tilde{p}:=({\tilde{𝐱}}^{*},\tilde{\theta },\hat{𝐮})\in \Omega_{h}^{t_{0}}$ and $Y^{\left(\leq \nu \right)}(t_{0},\tilde{p})(t_{0})=Y^{\left(\leq \nu \right)}(0,\hat{p})(t_{0})$. Since each *a*_*i*_ belongs to $\mathbb{C}\{𝐮\}[{𝐲}^{\left(\leq \nu \right)}]$, it follows from Lemma 5.11 that $a_{i}(t_{0},\tilde{p})(t_{0})=a_{i}(0,\hat{p})(t_{0})$ for all *i*. Applying Lemma 5.11 to the equation P(h)=0, we find that $\sum_{i=1}^{k}a_{i}(0,\hat{p})(t_{0})h(t_{0},\tilde{p})(t_{0}{)}^{i}=0$. Thus $h(t_{0},\tilde{p})(t_{0})$ is a root of the non-zero polynomial $\sum_{i=1}^{k}a_{i}(0,\hat{p})(t_{0})Z^{i}$. Noting that $h({\tilde{𝐱}}^{*},\tilde{\theta })=h(t_{0},\tilde{p})(t_{0})$ we conclude that $S_{h}(t_{0},\hat{p})$ is finite.

Part 2: ⇒ Assume that *h* is not algebraic over $\mathbb{C}\langle 𝐮\rangle ({𝐲}^{\left(\leq \nu \right)})$ and that *h* is ν-identifiable. We will give a proof by contradiction. The proof can be divided into four steps:

**Step 0** Label the rings that will be used in the proof.

**Step 1** Choose $\hat{p}=({\hat{𝐱}}^{*},\hat{\theta },\hat{𝐮})$. This will be done in terms of the non-vanishing of minimal polynomials over fraction fields of intermediate rings. Fix *V* and *t*_0_ and note that Lemma 5.11 with $\left(t_{1},t_{2},\hat{p})=(0,t_{0},\hat{p}\right)$ gives a ring homomorphism φ:R[h]→ℂ.

**Step 2** Show that the set $\left\{\delta (h)\;|\;\delta :R[h]\to \mathbb{C}\text{ is a }\mathbb{C}\text{-algebra homomorphism such that }\delta ({𝐮}^{\left(\mathbb{N}\right)})=\varphi ({𝐮}^{\left(\mathbb{N}\right)})\text{ and }\delta ({𝐲}^{\left(\leq \nu \right)})=\varphi ({𝐲}^{\left(\leq \nu \right)})\right\}$ is infinite. This will involve careful extension of ring homomorphisms.

**Step 3** Verify that $S_{h}(t_{0},\hat{p})$ is infinite by noting that each δ corresponds to a tuple $(\delta (𝐱),\delta (\theta ),\hat{𝐮})\in \Omega_{h}^{t_{0}}$.

We begin the proof.

**Step 0** Label $x_{1},\ldots ,x_{n},\theta_{1},\ldots ,\theta_{\ell }$ as $b_{1},\ldots ,b_{n+\ell }$. Without loss of generality, let *k* be such that j≤k if and only if *b*_*j*_ is algebraic over $\mathbb{C}\langle 𝐮\rangle ({𝐲}^{\left(\leq \nu \right)})$. Let *R*_2_ equal $\mathbb{C}\{𝐮\}[{𝐲}^{\left(\leq \nu \right)},b_{1},\ldots ,b_{k}]$ and let *F*_2_ be its field of fractions. Let μ be such that $b_{k+1},\ldots ,b_{\mu },h$ is a transcendence basis for Frac(R)=ℂ⟨𝐮⟩(𝐱,θ) over *F*_2_, relabeling if necessary. Let $R_{3}=R_{2}[b_{k+1},\ldots ,b_{\mu }]$ and let *F*_3_ be its field of fractions. Now $b_{\mu +1},\ldots ,b_{n+\ell }$ are each algebraic over $F_{4}:=F_{2}(b_{k+1},\ldots ,b_{\mu },h)$. For j=μ+1,…,n+ℓ let *P*_*j*_(*Z*) be the minimal polynomial of *b*_*j*_ over *F*_4_ multiplied by the LCM of the denominators, where *F*_4_ is viewed as the field of fractions of $R_{4}:=R_{2}[b_{k+1},\ldots ,b_{\mu },h]$. For each j=μ+1,…,n+ℓ, let $L_{j}(b_{k+1},\ldots ,b_{\mu },h)\in R_{4}$ be the leading coefficient of *P*_*j*_. Write $h=n_{h}/d_{h}$, where $n_{h},d_{h}\in R$. Let $P_{Qd_{h}}(Z)$ be the minimal polynomial of $Q\cdot d_{h}$ over *F*_4_ multiplied by the LCM of the denominators. (Note that if $Qd_{h}\in F_{4}$ then $P_{Qd_{h}}(Z)=Z-Qd_{h}$.) Let $L_{Qd_{h}}(b_{k+1},\ldots ,b_{\mu },h)$ and $C_{Qd_{h}}(b_{k+1},\ldots ,b_{\mu },h)$ be its leading and constant coefficients, respectively.

**Table 1:** Intermediate rings and their fraction fields.

Frac(R)=	ℂ⟨𝐮⟩(𝐱,θ)	*R*[*h*] =	ℂ{𝐮}[𝐱,θ,h](=ℂ[θ,h]{𝐮,𝐱,𝐲}/𝒥)
*F*_4_ =	⋮		⋮
	$F_{4}(Qd_{h})$		$R_{4}[Qd_{h},b_{\mu +1},b_{\mu +2}]$
			$R_{4}[Qd_{h},b_{\mu +1}]$
			$R_{4}[Qd_{h}]$
	*F*_3_(*h*)	*R*_4_ :=	*R*_3_[*h*]
*F*_3_ =	$F_{2}(b_{k+1},\ldots ,b_{\mu })$	*R*_3_ :=	$R_{2}[b_{k+1},\ldots ,b_{\mu }]$
*F*_2_ =	$\mathbb{C}\langle 𝐮\rangle \left({𝐲}^{\left(\leq \nu \right)},b_{1},\ldots ,b_{k}\right)$	*R*_2_ :=	$\mathbb{C}\{𝐮\}\left[{𝐲}^{\left(\leq \nu \right)},b_{1},\ldots ,b_{k}\right]$

**Step 1** Let Ψ and *U* be as guaranteed by the definition of ν-identifiability. Consider the subset of $\Omega_{h}^{0}$ consisting of all $\hat{p}$ such that for all j=μ +1,…,n+ℓ−1 the expressions *L*_*j*_, $L_{Qd_{h}}$, and $C_{Qd_{h}}$ evaluated at $\hat{p}$ and then *t* = 0 are not zero. This subset is non-empty because it is the intersection of finitely many non-empty Zariski open sets. Hence its intersection with Ψ×U is non-empty. Fix such a $\hat{p}=({\hat{𝐱}}^{*},\hat{\theta },\hat{𝐮})$. Let *V* be as required by the definition of ν-identifiability, let $t_{0}\in V\cap D_{h}(0,\hat{p})$ (recall Notation 5.3), and let φ be the ℂ-algebra homomorphism from *R*[*h*] to ℂ given by applying the ring homomorphism described in Lemma 5.11 with $\left(t_{1},t_{2},\hat{p})=(0,t_{0},\hat{p}\right)$.

**Step 2** We now show that there are infinitely many c∈ℂ such that there exists a ℂ-algebra homomorphism δ:R[h]→ℂ such that $\delta ({𝐮}^{\left(\mathbb{N}\right)})=\varphi ({𝐮}^{\left(\mathbb{N}\right)})$, $\delta ({𝐲}^{\left(\leq \nu \right)})=\varphi ({𝐲}^{\left(\leq \nu \right)})$, and δ(h)=c.

Define δ on *R*_2_ by $\delta :=\varphi {|}_{R_{2}}$. For each *j*, define $\delta (L_{j})(b_{k+1},\ldots ,b_{\mu },h)\in \mathbb{C}[b_{k+1},\ldots ,b_{\mu },h]$ to be the element obtained by evaluating the coefficients (in *R*_2_) of *L*_*j*_ via δ; define the analogous expressions with *L*_*j*_ replaced by $L_{Qd_{h}}$ and $C_{Qd_{h}}$. By the way $\hat{p}$ was chosen, we have that $\delta (L_{Qd_{h}})(b_{k+1},\ldots ,b_{\mu },h)$, $\delta (C_{Qd_{h}})(b_{k+1},\ldots ,b_{\mu },h)$, and all $\delta (L_{j})(b_{k+1},\ldots ,b_{\mu },h)$ are non-zero. Using Lemma 5.13 we can extend δ to $R_{3}:=R_{2}[b_{k+1},\ldots ,b_{\mu }]$ in a way that makes neither $\delta (L_{Qd_{h}})(\delta (b_{k+1}),\ldots ,\delta (b_{\mu }),h)$ nor $\delta (C_{Qd_{h}})(\delta (b_{k+1}),\ldots ,\delta (b_{\mu }),h)$ nor any $\delta (L_{j})(\delta (b_{k+1}),\ldots ,\delta (b_{\mu }),h)\in \mathbb{C}[h]$ equal to zero. Choose such an extension and call this, somewhat abusing notation, δ. Since *h* is not algebraic over *F*_3_, by Lemma 5.13 δ can be extended by mapping *h* to any element of ℂ. Now there are infinitely many *c* such that $\delta (L_{Qd_{h}})(\delta (b_{k+1}),\ldots ,\delta (b_{\mu }),c)\cdot \delta (C_{Qd_{h}})(\delta (b_{k+1}),\ldots ,\delta (b_{\mu }),c)\cdot \prod_{j}\delta (L_{j})(\delta (b_{k+1}),\ldots ,\delta (b_{\mu }),c)\neq 0$. Fix such a *c* for the remainder of the proof and extend δ to *R*_4_ by letting δ(h)=c.

We now extend δ to $R_{4}[Qd_{h}]$. By the preceding discussion, the leading and constant coefficients of $\delta (P_{Qd_{h}})(Z)$ are non-zero, so by Lemma 5.13 we can extend δ to $R_{4}[Qd_{h}]$ so that $\delta (Qd_{h})\neq 0$.

Next we extend δ to $R_{4}[Qd_{h},b_{\mu +1}]$. Observe that the minimal polynomial $Q_{\mu +1}(Z)$ of $b_{\mu +1}$ over $F_{4}(Qd_{h})$ is a factor of $P_{\mu +1}(Z)$. Therefore $\delta (Q_{\mu +1})(Z)$ is a non-zero factor of $\delta (P_{\mu +1})(Z)$. By Lemma 5.13 we can extend δ to $R_{4}[Qd_{h},b_{\mu +1}]$.

The argument from the preceding paragraph can be repeated to show that δ can be extended to $R_{4}[Qd_{h},b_{\mu +1},b_{\mu +2}]$. Applying this several more times, we see that we can extend δ to *R*[*h*].

Let $T=\{\delta \;|\;\delta :R[h]\to \mathbb{C}\text{ is a }\mathbb{C}\text{-algebra homomorphism such that }\delta ({𝐮}^{\left(\mathbb{N}\right)})=\varphi ({𝐮}^{\left(\mathbb{N}\right)})\text{ and }\delta ({𝐲}^{\left(\leq \nu \right)})=\varphi ({𝐲}^{\left(\leq \nu \right)})\}$. We have shown that {δ(h)|δ∈T} is infinite.

**Step 3** We will now show that $S_{h}(t_{0},\hat{p})$ is infinite. We have


$$S_{h}(t_{0},\hat{p})⊇\{h(\delta (𝐱),\delta (\theta ))\;|\;\exists \delta \in T\;(\delta (𝐱),\delta (\theta ),\hat{𝐮})\in \Omega_{h}^{t_{0}}\text{ and }\;Y^{\left(\leq \nu \right)}(t_{0},\delta (𝐱),\delta (\theta ),\hat{𝐮})(t_{0})=Y^{\left(\leq \nu \right)}(0,{\hat{𝐱}}^{*},\hat{\theta },\hat{𝐮})(t_{0})\}.$$

By Lemma 5.11 it holds that $Y^{\left(\leq \nu \right)}(t_{0},\delta (𝐱),\delta (\theta ),\hat{𝐮})(t_{0})=\delta ({𝐲}^{\left(\leq \nu \right)})$ and $Y^{\left(\leq \nu \right)}(0,{\hat{𝐱}}^{*},\hat{\theta },\hat{𝐮})(t_{0})=\varphi ({𝐲}^{\left(\leq \nu \right)})$. Hence


$$S_{h}(t_{0},\hat{p})⊇\{\delta (h)\;|\;\exists \delta \in T\;(\delta (𝐱),\delta (\theta ),\hat{𝐮})\in \Omega_{h}^{t_{0}}\text{ and }\delta ({𝐲}^{\left(\leq \nu \right)})=\varphi ({𝐲}^{\left(\leq \nu \right)})\}.$$

Since for each δ∈T we have $\delta (Qd_{h})\neq 0$, the first conjunct is satisfied. By the definition of *T*, the second conjunct is satisfied for all δ∈T. Hence


$$S_{h}(t_{0},\hat{p})⊇\{\delta (h)\;|\;\delta \in T\}.$$

We showed in Step 2 that the right hand side is infinite, and thus $S_{h}(t_{0},\hat{p})$ is infinite. Therefore *h* is not ν-identifiable, contradicting our assumption. ◻

**Lemma 5.13.** *Let W be a (possibly infinite) set of indeterminates and let S be a ℂ-subalgebra of the field of rational functions ℂ(W). Let φ:S→ℂ be a ℂ-algebra homomorphism. Let f∈ℂ(W)\{0}.*

*Suppose f is not algebraic over Frac(S). Then φ can be extended to S[f] by mapping f to any element of ℂ.*

*Suppose f is algebraic over Frac(S). Let P(Z) be the minimal polynomial of f over Frac(S) multiplied by the LCM of the denominators. Write $P(Z)=a_{k}Z^{k}+\ldots +a_{0}$, where $a_{k},\ldots ,a_{0}\in S$. If $\varphi (a_{k})\neq 0$, then φ can be extended to S[f]. If furthermore $\varphi (a_{0})\neq 0$, then in this extension φ(f)≠0.*

*Proof*: Suppose *f* is not algebraic over Frac(S). Our result follows from [2, p. 99].

Suppose *f* is algebraic over Frac(S) and $\varphi (a_{k})\neq 0$. If f∈Frac(S) then the result is trivial. Assume *f* is not in Frac(S). By [2, Theorem 3.2 p. 347], φ can be extended to *S*[*f*] or $\text{S}[{\text{f}}^{-1}]$. If the former is true the proof is complete. Suppose φ can be extended to $\varphi_{1}:S[f^{\;-1}]\to \mathbb{C}$. Writing $P(Z)=\sum_{i=0}^{k}a_{i}Z^{i}$, we have that $\sum_{i=0}^{k}a_{i}(f^{\;-1}{)}^{k-i}=0$. If $\varphi_{1}(f^{\;-1})=0$, then it follows that $\varphi_{1}\left(\sum_{i=0}^{k}a_{i}(f^{\;-1}{)}^{k-i}\right)=\varphi_{1}(a_{k})=0$, which contradicts our hypotheses. Thus $\varphi_{1}(f^{\;-1})\neq 0$. Now we can extend $\varphi_{1}$ to the ring $S[f^{\;-1}{]}_{\ker\varphi_{1}}$, which is equal to the subring of ℂ(W) consisting of fractions with numerators in $\text{S}[{\text{f}}^{-1}]$ and denominators in $S[f^{\;-1}]\backslash \ker\varphi_{1}$ (cf. [2, p. 346]). Since $f^{\;-1}\notin \ker\varphi_{1}$, the element *f* belongs to $S[f^{\;-1}{]}_{\ker\varphi_{1}}$. By restricting $\varphi_{1}$ to *S*[*f*] we have an extension of φ to *S*[*f*].

Still assuming *f* is algebraic over Frac(S), suppose $\varphi (a_{0})\neq 0$. The image of *f* must satisfy $\sum_{i=0}^{k}\varphi (a_{i})\varphi (f{)}^{i}=0$. Thus it is impossible that φ(f)=0. ◻

It is not always obvious whether a given field element is algebraic over a given subfield. The following proposition gives an equivalence that, for our purposes, reduces this question to the problem of checking the rank of a matrix with easily computable entries. We will use the following notation:

**Notation 5.14.** *Let $W=W_{1},\ldots ,W_{a}$ be elements of a ℂ-algebra and let $Z=Z_{1},\ldots ,Z_{b}$ be an algebraically independent set over ℂ. We denote by $\frac{\partial W}{\partial Z}$ the matrix whose (i,j)-th entry is $\frac{\partial W_{i}}{\partial Z_{j}}$. If M is such a matrix and $Z_{0}\in Z$, then $M\backslash Z_{0}$ denotes the result when the column corresponding to Z_0_ is removed. If this column does not appear in M then $M\backslash Z_{0}$ is equal to M.*

We will use the following algebraic fact, which generalizes [12, Thm. 2.3].

**Proposition 5.15.** *Let $p_{1},\ldots ,p_{s}$ each be algebraic over $\mathbb{C}(w_{1},\ldots ,w_{a},z_{1},\ldots ,z_{b})$, where s≤b and all w_i_ and z_i_ are indeterminates. The elements $p_{1},\ldots ,p_{s}$ are algebraically independent over $\mathbb{C}(w_{1},\ldots ,w_{a})$ if and only if the matrix $\partial (p_{1},\ldots ,p_{s})/\partial (z_{1},\ldots ,z_{b})$ has rank equal to s.*

*Proof*: Let $M:=\partial (p_{1},\ldots ,p_{s})/\partial (z_{1},\ldots ,z_{b})$ and let $M_{1}:=\partial (w_{1},\ldots ,w_{a},p_{1},\ldots ,p_{s})/$ $\partial (w_{1},\ldots ,w_{a},z_{1},\ldots ,z_{b})$. Note that


$$M_{1}=(\begin{array}{cc} I & 0\\ A & M \end{array}),$$

where *I* is the a×a identity matrix and *A* is an s×a matrix.

Now $p_{1},\ldots ,p_{s}$ is algebraically independent over $\mathbb{C}(w_{1},\ldots ,w_{a})$ iff $w_{1},\ldots ,w_{a},p_{1},\ldots ,p_{s}$ is algebraically independent over ℂ iff (by [12, Thm. 2.3]) $\text{rank}M_{1}=a+s$ iff rankM=s. ◻

**Proposition 5.16.** *Let $\nu \in {\mathbb{N}}^{m}$ and let h∈ℂ(𝐱,θ). Then h is algebraic over $\mathbb{C}\langle 𝐮\rangle ({𝐲}^{\left(\leq \nu \right)})$ if and only if the matrix $\frac{\partial ({𝐲}^{\left(\leq \nu \right)})}{\partial (𝐱,\theta )}$ has the same rank as $\frac{\partial ({𝐲}^{\left(\leq \nu \right)},h)}{\partial (𝐱,\theta )}$.*

*Proof*: Note that a subset of ${𝐲}^{\left(\leq \nu \right)}\cup \{h\}$ is algebraically independent over ℂ⟨𝐮⟩ if and only if it is algebraically independent over $\mathbb{C}({𝐮}^{\left(\leq \nu_{max}\right)})$. Let *T* be a maximal subset of ${𝐲}^{\left(\leq \nu \right)}$ that is algebraically independent over ℂ⟨𝐮⟩. Then by Proposition 5.15 with $w_{1},\ldots ,w_{a}={𝐮}^{\left(\leq \nu_{max}\right)}$ and $z_{1},\ldots ,z_{b}=𝐱\cup \theta$ we have $\text{rank}\frac{\partial (T)}{\partial (𝐱,\theta )}=\text{rank}\frac{\partial ({𝐲}^{\left(\leq \nu \right)})}{\partial (𝐱,\theta )}=|T|$.

Suppose *h* is algebraic over $\mathbb{C}\langle 𝐮\rangle ({𝐲}^{\left(\leq \nu \right)})$. Then T∪{h} is algebraic over $\mathbb{C}({𝐮}^{\left(\leq \nu_{max}\right)})$. Then $\text{rank}\frac{\partial ({𝐲}^{\left(\leq \nu \right)},h)}{\partial (𝐱,\theta )}=\text{rank}\frac{\partial (T,h)}{\partial (𝐱,\theta )}<|T|+1$ by Proposition 5.15. Therefore $\text{rank}\frac{\partial ({𝐲}^{\left(\leq \nu \right)},h)}{\partial (𝐱,\theta )}=|T|$.

Suppose *h* is not algebraic over $\mathbb{C}\langle 𝐮\rangle ({𝐲}^{\left(\leq \nu \right)})$. Then T∪{h} is not algebraic over $\mathbb{C}({𝐮}^{\left(\leq \nu_{max}\right)})$. Then $\text{rank}\frac{\partial ({𝐲}^{\left(\leq \nu \right)},h)}{\partial (𝐱,\theta )}=\text{rank}\frac{\partial (T,h)}{\partial (𝐱,\theta )}=|T|+1$ by Proposition 5.15. ◻

**Corollary 5.17.** *Algorithm 1 always terminates. The output is “Yes” if and only if h is ν-identifiable.*

*Proof*: Termination is obvious. The other result follows from Proposition 5.12 and Proposition 5.16. ◻

**Proposition 5.18.** *Let $\nu \in {\mathbb{N}}^{m}$ and let J be as in Algorithm 2. Let z∈𝐱∪θ. Then z is algebraic over $\mathbb{C}\langle 𝐮\rangle ({𝐲}^{\left(\leq \nu \right)})$ if and only if rank(J\z)=rank(J)−1.*

*Proof*: Without loss of generality we assume $z=\theta_{\ell }$. By Proposition 5.16, $\theta_{\ell }$ is algebraic over $\mathbb{C}\langle 𝐮\rangle ({𝐲}^{\left(\leq \nu \right)})$ if and only if $\text{rank}J=\text{rank}\frac{\partial ({𝐲}^{\left(\leq \nu \right)},\theta_{\ell })}{\partial (𝐱,\theta )}$. Now


$$\text{rank}\frac{\partial ({𝐲}^{\left(\leq \nu \right)},\theta_{\ell })}{\partial (𝐱,\theta )}=\text{rank}(\begin{array}{c} J\\ \left(0,\ldots ,0,1\right) \end{array})=\text{rank}(\begin{array}{cc} J\backslash \theta_{\ell } & 0\\ 0 & 1 \end{array})=\text{rank}(J\backslash \theta_{\ell })+1.$$

◻

**Corollary 5.19.** *Algorithm 2 always terminates. The output is “Yes” if and only if $\theta_{i}$ is ν-identifiable.*

*Proof*: Termination is obvious. The other result follows from Proposition 5.12 and Proposition 5.18. ◻

### 5.3 Probabilistic method for improved speed

#### 5.3.1 Presentation of algorithms and summary of results.

Algorithm 1 and 2 involve computing the rank of a matrix of rational expressions. It is usually much faster to insert random numbers for the variables and compute the rank of the resulting matrix. The disadvantage of this is that the numerical matrix may have lower rank than the symbolic. In [28] a method for doing this in the case where the coefficients of Σ are integers with user-specified probability of success was given. We adapt that method to our algorithms.

For the rest of this section, assume the coefficients of **f** and **g** in (2) and *h* in Algorithm 1 are integers. Note that the entries of *J* belong to $\mathbb{Q}(𝐱,\theta ,𝐮,{𝐮}^{\prime },\ldots ,{𝐮}^{\left(\nu_{max}\right)})$. Our strategy involves randomly choosing $n+\ell +(\nu_{max}+1)r$ non-negative integers and a prime number, and then evaluating the determinant of our matrix at these integers modulo the prime. Algorithm 3 implements this strategy on Algorithm 1.

**Algorithm 3 Determines whether parameter combination *h* is ν-identifiable.**

**Input** : Equations ${𝐱}^{\prime }=𝐟(𝐱,\theta ,𝐮),\;𝐲=𝐠(𝐱,\theta ,𝐮)$, where $(𝐟,𝐠)\notin {\mathbb{C}}^{n+m}$

$\nu \in {\mathbb{N}}^{m}$

h∈ℚ(𝐱,θ)

α∈(0,1)

**Output** : “Yes” if *h* is ν-identifiable with probability at least α

“No” if *h* is not ν-identifiable with probability at least α

Step 1a: Compute the least μ∈ℕ such that $2-(1-1/\mu {)}^{-2}\geq \alpha$.

Step 1b: Compute *D* and the least *N* as in Proposition 5.25 with $m^{*}=m+1$.

Step 1c: Choose $(T,p)\in \{0,1,2,\ldots ,\mu D{\}}^{n+\ell +(\nu_{max}+1)r}\times \left\{p\text{ prime }\;|\;N+1\leq p\leq 2N\right\}$ uniformly at random.

Step 1d: If *Q*(*T*) ≡ 0 mod *p*, repeat Step 1c. Otherwise continue to Step 1e.

Step 1e: Compute *J*_*h*_(*T*) mod *p*.

Step 2: Compute $\text{rank}\left(J_{h}(T)\mathrm{mod}p\right)$ and rank (*J*(*T*) mod *p*).

Step 3: If $\text{rank}\left(J_{h}(T)\mathrm{mod}p\right)=\text{rank}\left(J(T)\mathrm{mod}p\right)$, output “Yes”. Otherwise output “No”.

**Algorithm 4 Determines the ν-identifiable subset of 𝐱∪θ.**

**Input** : Equations ${𝐱}^{\prime }=𝐟(𝐱,\theta ,𝐮),\;𝐲=𝐠(𝐱,\theta ,𝐮)$, where $(𝐟,𝐠)\notin {\mathbb{C}}^{n+m}$

$\nu \in {\mathbb{N}}^{m}$

α∈(0,1)

**Output** : Subset of 𝐱∪θ consisting of exactly the ν-identifiable elements, with

probability at least α

Step 1a: Compute the least μ∈ℕ such that $2-(1-1/\mu {)}^{-2}\geq \alpha$.

Step 1b: Compute *D* and the least *N* as in Proposition 5.25 with $m^{*}=m$.

Step 1c: Choose $(T,p)\in \{0,1,2,\ldots ,\mu D{\}}^{n+\ell +(\nu_{max}+1)r}\times \left\{p\text{ prime }\;|\;N+1\leq p\leq 2N\right\}$ uniformly at random.

Step 1d: If Q(T)≡0modp, repeat Step 1c. Otherwise continue to Step 1e.

Step 1e: Compute *J*(*T*) mod *p*.

Step 2: Compute rank(J(T)modp).

Step 3: Let $S_{out}=\emptyset$.

For z∈𝐱∪θ

Compute rank(J(T)\zmodp).

If rank(J(T)\zmodp)<rank(J(T)modp), add *z* to *S*_*out*_.

End For

Step 4: Return *S*_*out*_.

While Algorithm 2 determines whether an individual parameter is ν-identifiable, Algorithm 4 uses this concept to determine, with user-specified probability, all elements of 𝐱∪θ that are ν-identifiable.

The main results on Algorithm 3 and Algorithm 4 are the following:

1. The expected time to reach Step 1e is negligible. Once Step 1e is reached the algorithm is guaranteed to terminate. (Proposition 5.29)
2. The probability that the output is correct is at least α. (Propositions 5.30 and 5.31)
3. For Algorithm 3, if in Step 2 rank(J(T)modp)=n+ℓ, then *h* is ν-identifiable. For Algorithm 4, if in Step 2 rank(J(T)modp)=n+ℓ, then every element of 𝐱∪θ is ν-identifiable. (Proposition 5.32)

**Remark 5.20.** *At the beginning of this section, we assumed n, m, and r are positive integers. As noted in Example 5.5, ℓ and r must be at least equal to the number of parameters and outputs, respectively, that appear in Σ, but can be chosen to be greater without changing identifiability results. In Algorithms 5.3.1 and 5.3.1 it is sufficient to use ℓ and r equal to the number of parameters and inputs, respectively, appearing in the equations. In particular one can use *r* = 0 if no inputs appear and the main results on the algorithms are correct.*

**Remark 5.21.** *We have presented Algorithms 5.3.1 and 5.3.1 only for the case where* ***f*** *and* ***g*** *are not all elements of ℂ, since removing this restriction would require addressing special cases in several of the proofs. If $(𝐟,𝐠)\in {\mathbb{C}}^{n+m}$, then h is ν-identifiable if and only if h∈ℂ. One could easily add a step at the beginning of either algorithm to accommodate this case.*

**Remark 5.22.** *Our algorithms do not specify the methods used to compute the ranks of matrices. One can use the state-of-the-art method for this to achieve maximum speed.*

#### 5.3.2 Proof of algorithms.

In Algorithms 5.3.1 and 5.3.1, a random tuple of integers *T* and a random prime *p* are chosen, we check that the denominator of a determinant evaluated at *T* does not vanish modulo *p*, and then calculate the rank of a matrix after evaluating at *T* modulo *p*. We show that this gives the correct results with user-specified probability. The main results on Algorithms 3 and 5.3.1 are Propositions 5.29, 5.30, 5.31, and 5.32. The theory is based on bounds on integer roots of polynomials with integer coefficients, as well as the distribution of the prime numbers.

First, we use Proposition 5.23, Lemma 5.24, and Lemma 5.26 to prove Proposition 5.25, which gives conditions on the sets from which we choose *T* and *p* so that the numerator of the determinant evaluated at *T* does not vanish modulo *p* with user-specified probability. This proposition is essentially a more precisely stated version of [28, Proposition 6], and the proof we give is outlined in [28].

Next, Proposition 5.28 shows that the probability that the numerator vanishes given that the denominator does not vanish can be specified by the user. Note that this is the true probability associated with the algorithm, since we must first check that our choice of (*T*,*p*) does not make the denominator vanish before proceeding. This issue is not addressed in similar algorithms (cf. [29, p. 739], [17]) and is non-trivial, as shown by Example 5.27.

Finally, we prove statements about the algorithms that are directly relevant to helping the user interpret them. Although in principle, arbitrarily many instances of (*T*,*p*) may need to be chosen before finding one that does not make the denominator vanish, Proposition 5.29 shows that the expected time for a successful choice is negligible. It also asserts the algorithm’s termination after such a successful choice. Propositions 5.30 and 5.31 show that the algorithms produce the correct result with user-specified probability. Proposition 5.32 states that when the rank of the specialized matrix is full, the algorithms output the correct result with certainty.

**Proposition 5.23** ([3] Prop. 98 p. 192). *Let $P(Z_{1},\ldots ,Z_{k})$ be a polynomial of total degree D over an integral domain A. Let S⊆A. If an element $\left(z_{1},\ldots ,z_{k}\right)$ is chosen from ${\text{S}}^{\text{k}}$ uniformly at random, then*


$$\text{prob}(P(z_{1},\ldots ,z_{k})=0)\leq \frac{D}{|S|}.$$

**Lemma 5.24** ([34] Lemma 18.9 p. 525). *Let S⊆ℕ be a nonempty finite set of prime numbers, let M∈ℤ\{0}, and let C≥|M|. If p is chosen from S uniformly at random, then*


$$\text{prob}(p\text{ divides }M)\leq \frac{\ln C}{|S|\ln\min S}.$$

**Proposition 5.25.** *Let J (resp. J_h_) be as in Algorithm 2 (resp. Algorithm 1) and suppose J (resp. J_h_) has at least one non-zero entry. Let*

1.
$\mu \in {\mathbb{N}}^{+}$

2. *d*_*1*_ *=  maximum degree of the numerators and denominators of 𝐟 and 𝐠 (resp. 𝐟, 𝐠 and h)*
3.
$d_{2}=\max\{\ln(|a|+1):a\text{ is a coefficient in }𝐟\text{ or }𝐠\;(\text{resp. }𝐟\text{, }𝐠\text{, or }h)\}$

4. $D=(n+\ell )(2\nu_{max}+3)(n+m^{*})d_{1}$, *  where $m^{*}=m$ (resp. $m^{*}=m+1$)*
5. *C be such that $\ln C=(2\ln(n+\ell +r+1)+\ln(\mu D+1))D+(n+\ell )(2\nu_{max}+3)((n+m^{*})d_{2}+\ln(2nD))$,*
  where $m^{*}=m$ (resp. $m^{*}=m+1$)
6. N∈ℕ *such that N≥2μlnC*
7.
S={p prime |N+1≤p≤2N}

*Let J_0_ be a square submatrix of J (resp. J_h_) with rank equal to that of J (resp. J_h_). Note that the numerator of $\text{det }J_{0}$ lies in $\mathbb{Z}[𝐱,\theta ,𝐮,\ldots ,{𝐮}^{\left(\nu_{max}\right)}]$. If a tuple of values T of the variables is chosen uniformly at random from $\{0,1,2,\ldots ,\mu D{\}}^{n+\ell +(\nu_{max}+1)r}$ and p is chosen uniformly at random from S, then*


prob(p does not divide num det J0(T))≥(1−1μ)2,

*where $\text{num det }J_{0}(T)$ represents the specialization of the numerator of $\text{det }J_{0}$ at the chosen values.*

*Proof*: We prove the main version first. The version for *J*_*h*_ will follow quickly from this.

Note that since *J* has at least one non-zero entry, *d*_1_ is well-defined and positive, and hence *D* is positive. Let *W* denote $\text{num det }J_{0}(T)$. We have that

prob(p does not divide W)=prob(W≠0)·prob(p does not divide W given W≠0). (5)

By Proposition 5.23, we have that


$$\text{prob}(W=0)\leq \frac{\deg\text{num det }J_{0}}{\mu D+1}.$$

We show that the degree of the numerator of any entry of *J* is no greater than $(2\nu_{max}+3)(n+m)d_{1}$. Fix j∈{1,…,m}. We first show by induction that for k∈ℕ we can write $y_{j}^{\left(k\right)}=A_{k}/Q^{2k+1}$, where $\deg A_{k}\leq (2k+1)(n+m)d_{1}$. For the base case *k* = 0, recall from Notation 5.1 we have that $y_{j}=G_{j}/Q$, and $\deg G_{j}\leq (n+m)d_{1}$. For the inductive hypothesis, fix k≥0 and assume $y_{j}^{\left(k\right)}=A_{k}/Q^{2k+1}$ with $\deg A_{k}\leq (2k+1)(n+m)d_{1}$. Applying the quotient rule we have that

$$y_{j}^{\left(k+1\right)}=\frac{1}{Q^{4k+2}}\left(Q^{2k+1}\sum_{i=1}^{n}\frac{\partial A_{k}}{\partial x_{i}}\frac{F_{i}}{Q}-A_{k}(2k+1)Q^{2k}\sum_{i=1}^{n}\frac{\partial Q}{\partial x_{i}}\frac{F_{i}}{Q}\right),$$ (6)

recalling from Notation 5.1 that each $f_{i}=F_{i}/Q$. Since $\deg F_{i}$ and degQ do not exceed (*n* + *m*)*d*_1_, we conclude the inductive step. Continuing the proof of the bound on the degrees of the numerators of the entries of *J*, note that for any z∈𝐱∪θ we can write $\frac{\partial }{\partial z}(y_{j}^{\left(k\right)})=\frac{1}{Q^{4k+2}}(Q^{2k+1}\frac{\partial A_{k}}{\partial z}-A_{k}(2k+1)Q^{2k}\frac{\partial Q}{\partial z})$ where $\deg A_{k}\leq (2k+1)(n+m)d_{1}$. So we can write $\frac{\partial }{\partial z}(y_{j}^{\left(k\right)})=A/Q^{2k+2}$ where $\deg A\leq (2k+2)(n+m)d_{1}$. In *J*_0_ the value of *k* does not exceed $\nu_{max}$ so we conclude the argument.

Since *J*_0_ is square it has at most n+ℓ rows. Therefore $\deg\text{num det }J_{0}$ is no greater than *D*. Thus, we have

$$\text{prob}(W=0)\leq \frac{D}{\mu D+1}\leq \frac{1}{\mu }.$$ (7)

Assume that W≠0. Lemma 5.26 below shows that C≥|W|. Our assumptions imply that lnC≥3 and hence N≥6. From [34, Exercise 18.18] it follows that *S* is non-empty. Using Lemma 5.24 with *M* = *W*, we have


$$\text{prob}(p\text{ divides }W)\leq \frac{\ln C}{|S|\ln\min S}\leq \frac{\ln C}{|S|\ln N}.$$

By [34, Exercise 18.18], we have |S|>N/(2lnN). It follows that

$$\text{prob}(p\text{ divides }W)\leq \frac{2\ln C}{N}\leq \frac{2\ln C}{2\mu \ln C}=\frac{1}{\mu }.$$ (8)

Combining (5), (7), and (8), we have our result.

We now prove the version with *J*_*h*_. Let $\Sigma_{1}$ be the system obtained by adding the equation *y*_*m* + 1_ = *h* to Σ and set $\nu_{m+1}=0$. Now *J*_*h*_ for Σ is equal to *J* for $\Sigma_{1}$. Our result follows from the main version of the proposition. ◻

**Lemma 5.26.** *In the setup of Proposition 5.25, lnC≥ln|W|.*

*Proof*: For a polynomial *p* with integer coefficients, we shall define the height of *p* as ht(p):=max{ln(|a|+1)|a is a coefficient of p}. We will use the following properties ([28, Lemma 1]): For polynomials $p_{1},\ldots ,p_{s}$ in n+ℓ+r variables, tuple of integers *T*_0_, and partial derivative ∂, the following hold:

1. $\text{ht}(\partial p_{1})\leq \text{ht}(p_{1})+\ln\deg p_{1}$,
2. $\text{ht}(p_{1}+\ldots +p_{s})\leq \max\{\text{ht}(p_{1}),\ldots ,\text{ht}(p_{s})\}+\ln s$,
3. $\text{ht}(p_{1}p_{2})\leq \min\{\deg p_{1},\deg p_{2}\}\cdot \ln(n+\ell +r+1)+\text{ht}(p_{1})+\text{ht}(p_{2})$, and
4. $\text{ht}(p_{1}(T_{0}))\leq \max\{\text{ht}(t_{0})\;|\;t_{0}\in T_{0}\}\cdot \deg(p_{1})+\text{ht}(p_{1})$.

Fix j∈{0,…,m} and write $y_{j}^{\left(k\right)}=A_{k}/Q^{2k+1}$, we will prove by induction that for all k∈ℕ

$$\text{ht}(A_{k})\leq (2k+1)(n+m)\left(2d_{1}\ln(n+\ell +r+1)+d_{2}\right)\;\;+(k+1)\ln(2n(n+m)d_{1})+(2k+1)\ln(2k+1).$$ (9)

We noted earlier that degQ, all $\deg F_{i}$, and $\deg G_{j}$ are no greater than (*n* + *m*)*d*_1_. It follows from this and the product height property that $\text{ht}(Q),\text{ ht}(F_{i}),\text{ ht}(G_{j})\leq (n+m)\left(d_{1}\ln(n+\ell +r+1)+d_{2}\right)$. For conciseness, we will use $d_{1}^{*}$ and $d_{2}^{*}$ to denote these degree and height bounds, respectively.

For the base case *k* = 0, note that $A_{0}=G_{j}$. For the inductive hypothesis, fix k≥0 and suppose *A*_*k*_ satisfies (9). As shown in the (6), we have


$$A_{k+1}=\sum_{i=1}^{n}F_{i}\left(Q\cdot \frac{\partial A_{k}}{\partial x_{i}}-(2k+1)\cdot A_{k}\cdot \frac{\partial Q}{\partial x_{i}}\right).$$

Using the derivation and product height properties, as well as the bound $\deg A_{k}\leq (2k+1)d_{1}^{*}$, we have


$$\text{ht}\left(Q\cdot \frac{\partial A_{k}}{\partial x_{i}}\right)\leq d_{1}^{*}\ln(n+\ell +r+1)+d_{2}^{*}+\text{ht}(A_{k})+\ln\left((2k+1)d_{1}^{*}\right)$$

$$\text{and ht}\left((2k+1)\cdot A_{k}\cdot \frac{\partial Q}{\partial x_{i}}\right)\leq d_{1}^{*}\ln(n+\ell +r+1)+\ln(2k+2)+\text{ht}(A_{k})+d_{2}^{*}+\ln d_{1}^{*}.$$

By the sum height property we have that

$$\text{ht}\left(Q\cdot \frac{\partial A_{k}}{\partial x_{i}}-(2k+1)\cdot A_{k}\cdot \frac{\partial Q}{\partial x_{i}}\right)\leq d_{1}^{*}\ln(n+\ell +r+1)+d_{2}^{*}+\;\text{ht}(A_{k})+\ln\left((2k+2)d_{1}^{*}\right)+\ln2.$$ (10)

By the product height property we have

$$\text{ht}\left(F_{i}\cdot \left(Q\cdot \frac{\partial A_{k}}{\partial x_{i}}-(2k+1)\cdot A_{k}\cdot \frac{\partial Q}{\partial x_{i}}\right)\right)\leq d_{1}^{*}\ln(n+\ell +r+1)+(\text{RHS of }(10))+d_{2}^{*}.$$ (11)

By the sum height property we have

$$\text{ht}(A_{k+1})\leq 2d_{1}^{*}\ln(n+\ell +r+1)+2d_{2}^{*}+\text{ht}(A_{k})+\ln\left(2(2k+2)d_{1}^{*}\right)+\ln n\;=2(n+m)\left(2d_{1}\ln(n+\ell +r+1)+d_{2}\right)+\text{ht}(A_{k})\;\;+\ln\left(2n(n+m)d_{1}\right)+\ln(2k+2)\;\leq (2k+3)(n+m)\left(2d_{1}\ln(n+\ell +r+1)+d_{2}\right)+(k+2)\ln\left(2n(n+m)d_{1}\right)\;\;+(2k+1)\ln(2k+1)+\ln(2k+2).$$ (12)

Noting that (2k+1)ln(2k+1)+ln(2k+2)≤(2k+3)ln(2k+3), we conclude the inductive step.

Now for z∈𝐱∪θ, we have that $\frac{\partial }{\partial z}(y_{j}^{\left(k\right)})$ is equal to $\left(Q\cdot \frac{\partial A_{k}}{\partial z}-(2k+1)\cdot A_{k}\cdot \frac{\partial Q}{\partial z}\right)/Q^{2k+2}$. A bound on the height of the numerator is given by the right-hand side of (10). Since RHS of (10)  ≤  RHS of (11)  ≤  RHS of (12), we see that a bound on this height is also given by the RHS of (9) with *k* replaced by *k* + 1. Observing that the maximum value of *k* occurring for an entry in *J*_0_ is $\nu_{max}$ and applying the evaluation height property, we have that the numerator of each entry of *J*_0_(*T*) is bounded by


$$B:=(2\nu_{max}+3)(n+m)\left(\left(2\ln(n+\ell +r+1)+\ln(\mu D+1)\right)d_{1}+d_{2}\right)\;+(\nu_{max}+2)\ln\left(2n(n+m)d_{1}\right)+(2\nu_{max}+3)\ln(2\nu_{max}+3).$$

Since our bounds assume that entries in the same row have the same denominator, we have that $\text{num det }J_{0}(T)=\text{det }(\text{num }J_{0}(T))$, where $\text{num }J_{0}$ is the matrix whose elements are the numerators of *J*_0_. Henceforth we assume *J*_0_ has polynomial entries with heights bounded by *B* when evaluated at *T*. Using Hadamard’s Theorem, we have that $|W|\leq \prod_{i=1}^{\text{rank}J_{0}}||v_{i}||$, where $||v_{i}||$ is the square root of the sum of the squares of the elements of the *i*-th column of *J*_0_(*T*). Hence $||v_{i}||\leq \sqrt{n+\ell }\cdot e^{B}$. Thus


$$\ln|W|\leq (n+\ell )((2\nu_{max}+3)(n+m)((2\ln(n+\ell +r+1)+\ln(\mu D+1))d_{1}+d_{2})\;+(\nu_{max}+2)\ln\left(2n(n+m)d_{1}\right)+(2\nu_{max}+3)\ln(2\nu_{max}+3)+\frac{1}{2}\ln(n+\ell )).$$

Recalling that


$$\ln C=(2\ln(n+\ell +r+1)+\ln(\mu D+1))(n+\ell )(2\nu_{max}+3)(n+m)d_{1}\;\;+(n+\ell )(2\nu_{max}+3)((n+m)d_{2}+\ln(2n(n+\ell )(2\nu_{max}+3)(n+m)d_{1})),$$

we conclude the proof. ◻

After a set of random integers and a random prime number is chosen, we must first check that no denominator vanishes modulo the prime number before evaluating the rank of the matrix. Thus the probability that the algorithm gives the correct answer is not simply the probability that *p* does not divide $\text{num det }J_{0}(T)$, but rather the probability that *p* does not divide $\text{num det }J_{0}(T)$ given that *p* does not divide $\text{denomdet }J_{0}(T)$. If a random tuple *T* is chosen and used to evaluate two polynomials *A* and *B*, it is not necessarily the case that prob(A(T)≠0)≤prob(A(T)≠0 given B(T)≠0), as the following example shows:

**Example 5.27.** *Let k be a positive integer and let A(Z)=Z and B(Z)=(Z−1)(Z−2)·…·(Z−k). If an integer T is chosen uniformly at random from {0,1,2,…,k}, then $\text{prob}(A(T)\neq 0)=\frac{k}{k+1}$ and prob(A(T)≠0 given B(T)≠0)=0.*

The following proposition shows that the conditions used for Proposition 5.25 also give a bound on the conditional probability that is relevant to our algorithms.

**Proposition 5.28.** *Let J (resp. J_h_) be as in Algorithm 2 (resp. Algorithm 1) and suppose J (resp. J_h_) has at least one non-zero entry. Let μ, D, S, and J_0_ be as in Proposition 5.25. If a tuple of values T of the variables is chosen uniformly at random from $\{0,1,2,\ldots ,\mu D{\}}^{n+\ell +(\nu_{max}+1)r}$ and p is chosen uniformly at random from S, then*


prob(p does not divide  num det J0(T) given p does not divide Q(T))≥2−(1−1μ)−2,

*where $\text{num det }J_{0}(T)$ represents the specialization of the numerator of $\text{det }J_{0}$ at the chosen values.*

*Proof*: Let *M* denote the sample space $\{0,\ldots ,\mu D{\}}^{n+\ell +(\nu_{max}+1)r}\times S$. Let


$$A=\{(T,p)\in M\;|\;p\text{ divides num det }J_{0}(T)\},\;B=\{(T,p)\in M\;|\;p\text{ divides }Q(T)\}.$$

Now


$$\text{prob}((T,p)\notin A\text{ given }(T,p)\notin B)=\frac{\left|M|-|A|-|B|+|A\cap B\right|}{\left|M|-|B\right|}\geq 1-\frac{\left|A\right|}{\left|M|-|B\right|}.$$

By Proposition 5.25 we have $|A|/|M|\leq 1-(1-\frac{1}{\mu }{)}^{2}$. We now prove the same bound holds for |B|/|M|. Now $\deg Q\leq (n+m)d_{1}$, so using Proposition 5.23 we have prob(Q(T)=0)≤D/|S|=D/(μD+1)≤1/μ. By the first statement of [28, Prop. 3] with *j* = 0 we have that the height of *Q*(*T*) is no greater than $\left(n+m)(d_{2}+d_{1}(\ln(\mu D+1)+2\ln(n+\ell +r+1))\right)$, which is no greater than lnC. Thus C≥|Q(T)| and if Q(T)≠0 then by Lemma 5.24 we have prob(p divides Q(T))≤lnC/(|S|N), which by [34, Exercise 18.18] is no greater than 1/μ. Thus $|B|/|M|\leq 1-(1-\frac{1}{\mu }{)}^{2}$. Now we have


$$1-\frac{\left|A\right|}{\left|M|-|B\right|}\geq 1-\frac{1-{\left(1-\frac{1}{\mu }\right)}^{2}}{{\left(1-\frac{1}{\mu }\right)}^{2}}.$$

◻

**Proposition 5.29.** *Fix the input to Algorithm 3 (or Algorithm 4). (i) The probability that Step 1e will be reached in no more than k iterations of Step 1c is at least $1-(\frac{1-\alpha }{2-\alpha }{)}^{k}$. (ii) When Step 1e is reached the algorithm is guaranteed to terminate.*

*Proof*: (i) It was shown in the proof of Proposition 5.28 that $\text{prob}(Q(T)\equiv 0\mathrm{mod}p)\leq 1-(1-\frac{1}{\mu }{)}^{2}$, and based on the way μ was chosen in Step 1a it is trivial to verify that this is no greater than $\frac{1-\alpha }{2-\alpha }$. Therefore for *k* independent choices the probability that Q(T)≢0modp for at least one of them is at least $1-(\frac{1-\alpha }{2-\alpha }{)}^{k}$.

(ii) This is obvious. ◻

**Proposition 5.30.** *Fix the input to Algorithm 3. If h is ν-identifiable, the probability that the output is “Yes” is at least α. If h is not ν-identifiable, the probability that the output is “No” is at least α.*

*Proof*: Consider the following subsets of {(T,p)|Q(T)≢0modp}: Y={(T,p)|output is “Yes”}, N={(T,p)|output is “No”}, E={(T,p)|rank(J(T)modp)=rankJ}, and $E_{h}=\{(T,p)\;|\;\text{rank}(J_{h}(T)\mathrm{mod}p)=\text{rank}J_{h}\}$. We have


$$\text{prob}(Y)\geq \text{prob}(Y\text{ given }E)\text{prob}(E)\;\text{ and }\;\text{prob}(N)\geq \text{prob}(N\text{ given }E_{h})\text{prob}(E_{h}).$$

Proposition 5.28 gives us that prob(E)≥α and $\text{prob}(E_{h})\geq \alpha$.

Suppose *h* is ν-identifiable. By Propositions 5.12 and 5.16, we have $\text{rank}J_{h}=\text{rank}J$. We show that prob(Y given E)=1. Suppose (T,p)∈E. Then $\text{rank}(J_{h}(T)\mathrm{mod}p)\leq \text{rank}J_{h}=\text{rank}J=\text{rank}(J(T)\mathrm{mod}p)\leq \text{rank}(J_{h}(T)\mathrm{mod}p)$, and we deduce that $\text{rank}(J_{h}(T)\mathrm{mod}p)=\text{rank}(J(T)\mathrm{mod}p)$, so the algorithm outputs “Yes”.

Suppose *h* is not ν-identifiable. By Propositions 5.12 and 5.16, we have $\text{rank}J_{h}>\text{rank}J$. We show that $\text{prob}(N\text{ given }E_{h})=1$. Suppose $(T,p)\in E_{h}$. Then $\text{rank}(J_{h}(T)\mathrm{mod}p)=\text{rank}J_{h}>\text{rank}J\geq \text{rank}(J(T)\mathrm{mod}p)$, so the algorithm outputs “No”. ◻

**Proposition 5.31.** *Fix the input to Algorithm 4. Let S_id_ denote the ν-identifiable subset of 𝐱∪θ and let S_out_ denote the output of Algorithm 4. The probability that $S_{out}=S_{id}$ is at least α*.

*Proof*: Because *S*_*out*_ depends on (*T*,*p*) we shall use the notation *S*_*out*_(*T*,*p*). Consider the following subsets of {(T,p)|Q(T)≢0modp}: $Y=\{(T,p)\;|\;S_{out}(T,p)=S_{id}\}$ and E={(T,p)|rank(J(T)modp)=rankJ}. We have


prob(Y)≥prob(Y given E)prob(E).

By Proposition 5.28 we have prob(E)≥α. We show prob(Y given E)=1. Suppose (T,p)∈E. Let z∈𝐱∪θ. Suppose *z* is ν-identifiable. By Propositions 5.12 and 5.16, we have $\text{rank}J_{z}=\text{rank}J$. Now $\text{rank}\left(J_{z}(T)\mathrm{mod}p\right)\leq \text{rank}J_{z}=\text{rank}J=\text{rank}\left(J(T)\mathrm{mod}p\right)\leq \text{rank}\left(J_{z}(T)\mathrm{mod}p\right)$. Since the final row of *J*_*z*_(*T*) must be (0…1…0), it follows that $\text{rank}\left(J(T)\backslash z\mathrm{mod}p\right)=\text{rank}\left(J_{z}(T)\backslash z\mathrm{mod}p\right)=\text{rank}\left(J_{z}(T)\mathrm{mod}p\right)-1=\text{rank}\left(J(T)\mathrm{mod}p\right)-1$. Therefore $z\in S_{out}(T,p)$. Suppose *z* is not ν-identifiable. It follows from Propositions 5.12 and 5.16 that $\text{rank}J_{z}-1=\text{rank}J$. Because (T,p)∈E and the final row of *J*_*z*_(*T*) is (0…1…0) we have that $\text{rank}\left(J_{z}(T)\mathrm{mod}p\right)=\text{rank}J_{z}$. Now $\text{rank}\left(J(T)\backslash z\mathrm{mod}p\right)=\text{rank}\left(J_{z}(T)\backslash z\mathrm{mod}p\right)=\text{rank}(J_{z}(T)\mathrm{mod}p)-1=\text{rank}J_{z}-1=\text{rank}J$. Therefore $z\notin S_{out}(T,p)$. ◻

**Proposition 5.32.** *Suppose that in Step 2 of Algorithm 3 (resp. Algorithm 4) rank(J(T)modp)=n+ℓ. Then h is ν-identifiable and the output is “Yes” (resp. every element of 𝐱∪θ is ν-identifiable and the output is 𝐱∪θ).*

*Proof*: We first prove the statement regarding Algorithm 3. We have n+ℓ≥rankJ≥rank(J(T)modp), so rankJ=n+ℓ. It follows that $\text{rank}J_{h}=n+\ell$ and by Propositions 5.12 and 5.16 we have that *h* is ν-identifiable. Since $\text{rank}\left(J(T)\mathrm{mod}p\right)\leq \text{rank}\left(J_{h}(T)\mathrm{mod}p\right)\leq n+\ell$, it follows that $\text{rank}\left(J(T)\mathrm{mod}p\right)=\text{rank}\left(J_{h}(T)\mathrm{mod}p\right)$ and the output will be “Yes”.

We now address Algorithm 4. By the preceding paragraph rankJ=n+ℓ. Since for any z∈𝐱∪θ the matrix J\z has only n+ℓ−1 columns, it must be that rankJ\z<rankJ and hence by Propositions 5.12 and 5.18 each element of 𝐱∪θ is ν-identifiable. Similarly, rank(J(T)\zmodp)<rank(J(T)modp) and hence the output is 𝐱∪θ. ◻

## Supporting information

S1 File Maple code for algorithm 4. Currently contains entries for Drosophila period protein model.
(PDF)

S2 FileMaple code for algorithm 3.Currently contains entries for NF-κB model.(MW)

S3 Table Description of models. Details of the models used in the paper.
(MW)
