# Supplementary material for: A new method for a priori practical identifiability
Source: PLoS One. 2025 Jul 17;20(7):e0327593. doi: 10.1371/journal.pone.0327593 (PMC12270335; doi:10.1371/journal.pone.0327593)
Supplement: S1 File — Currently contains entries for Drosophila period protein model. (PDF) [file pone.0327593.s001.pdf]

A new method for a priori practical identifiability

1 Supplementary Material: Models used to illustrate our methods

1.1 Drosophila Period Protein [3]

$M' = \frac{v_s K_I^4}{K_I^4 + P_N^4} - \frac{v_m M}{K_m + M}$
$P_0' = k_s M - \frac{V_1 P_0}{K_1 + P_0} + \frac{V_2 P_1}{K_2 + P_1}$
$P_1' = \frac{V_1 P_0}{K_1 + P_0} + \frac{V_4 P_2}{K_4 + P_2} - P_1 \left(\frac{V_2}{K_2 + P_1} + \frac{V_3}{K_3 + P_1} \right)$
$P_2' = \frac{V_3 P_1}{K_3 + P_1} - P_2 \left(\frac{V_4}{K_4 + P_2} + k_1 + \frac{v_d}{K_d + P_2} \right) + k_2 P_N$
$P_N' = k_1 P_2 - k_2 P_N$
$y = P_N$

Table 1: States

State	Description
M	Concentration of Period (per) mRNA
P_0	Concentration of Unphosphorylated cytosolic PER
P_1	Concentration of Monophosphorylated cytosolic PER
P_2	Concentration of Bisphosphorylated cytosolic PER
P_N	Concentration of Nuclear biphosphorylated form of PER

Table 2: Parameters

Parameter	Description
ν_d	Maximum degradation rate of P_2 by enzyme
ν_m	Maximum rate of degradation of M by enzyme, in the cytosol
ν_s	Maximum accumulation rate for M in the cytosol
K_d	Michaelis constant of degradation of P_2 by enzyme
K_I	Threshold constant for repression
K_m	Michaelis constant, in cytosol, for degradation of M
k_1	First-order rate constant, for transport of P_2 into nucleus
k_2	First-order rate constant for transport of P_N into the cytosol
k_s	First-order rate constant, rate of synthesis of PER protein, proportional to M
V_i, $i \in \{1,2,3,4\}$	Maximum rate of kinase(s) and phosphatase(s) of P_0 into P_1 and P_1into P_2
K_i, $i \in \{1,2,3,4\}$	Michaelis constant of kinase(s) and phosphatase(s) of P_0 into P_1 and P_1into P_2

Remark: Degree of cooperativity (n) in [3] has been set to 4, i.e. $n = 4$.

Table 3: Input and Output

Variable	Description
y	Output

1.2 Model of the interaction between liver and pancreatic cells [2]

$$
\begin{aligned}
x_1' &= \frac{247x_2}{250} - \frac{247x_1}{250} + \frac{17\theta_1}{5000000} - \frac{17\theta_2 x_1}{1500} - \frac{340\theta_3 x_1 x_4}{9}\left(1 - \frac{\theta_6 x_5}{\theta_7 + x_5}\right)\\
x_2' &= \frac{247x_1}{250} - \frac{247x_2}{250}\\
x_3 &= \frac{247x_4}{250} - \frac{247x_3}{250} + \frac{100000000x_7\theta_4\theta_8 x_2^2}{\frac{3537}{50} + 100000000x_2^2}\\
x_4' &= \frac{247x_3}{250} - \frac{247x_4}{250} - \frac{17}{1500}\theta_5 x_4\\
x_5' &= \frac{10000x_1}{3} - \frac{11}{2}\\
x_6' &= \frac{20x_2}{3} - \frac{x_6}{500}\\
x_7' &= \left(-\frac{81}{2500000}x_6^2 + \frac{63}{100000}x_6 - \frac{1}{400}\right)\theta_9 x_7\\
y_1 &= \frac{5000}{3}(x_1 + x_2)\\
y_2 &= \frac{5000}{3}(x_4 + x_3)
\end{aligned}
$$

Table 4: States

State	Description
x_1	Number of glucose molecules in the liver
x_2	Number of glucose molecules in the pancreas
x_3	Number of insulin molecules in the pancreas
x_4	Number of insulin molecules in the liver
x_5	Represents the integral of excess glucose over time
x_6	Long-term average glucose concentration in the co-culture medium
x_7	β-cell volume

Table 5: Parameters

Parameter	Description
θ_1	Endogenous glucose production in the liver spheroids
θ_2	Insulin-independent glucose disposal rate
θ_3	Insulin sensitivity of liver spheroids
θ_4	Maximal insulin secretion rate per unit volume of β cells
θ_5	Hepatic insulin clearance
θ_6	Maximal fractional reduction of insulin sensitivity
θ_7	Value of time integral of excess glucose providing half of the maximal fractional reduction
θ_8	In-vitro functionality of liver islets
θ_9	Scaling parameter for the rate of change of β-cell number

Remark: We have replaced

$$1 - \frac{Vm_{f_{islets}} \cdot t^2}{\alpha^2 + t^2}$$

with θ_8.

Table 6: Input and Output

Variable	Description
y_1	Output
y_2	Output

Table 7: Known parameters

Known parameter	Value	Description
$V_{m,liver}$	$\frac{3}{10000}$	Volume of co-culture medium in the liver compartment
V_{hep}	$\frac{17}{5000000}$	Total volume of HepaRG cells in the MPS
$V_{m,pancreas}$	$\frac{3}{10000}$	Volume of co-culture medium in the pancreas compartment
Q	$\frac{741}{2500000}$	Flow rate between culture compartments
$G_{healthy}$	$\frac{11}{2}$	Average glucose value in health
$EC50_I$	$\frac{393}{50}$	Glucose concentration resulting in half-of-maximum response to insulin of the β cells
d_0	$\frac{1}{400}$	Rate of β-cell death at zero glucose
r_1	$\frac{63}{100000}$	Beta cell mass constant
r_2	$\frac{81}{2500000}$	Beta cell mass constant
τ_{slow}	500	Time constant for averaging the glucose signal
hep	1	Hepatocytes present in the 2-OC
$islets$	1	Islets present in the 2-OC

1.3 Model of insulin-uptake [1]

$IR' = -k_1 \cdot IR \cdot u - k_{1,basal} \cdot IR + k_R \cdot IR_i + IR_{ins} \cdot k_{m,1} + k_{m,2} \cdot IR_P$
$IR'_{ins} = k_1 \cdot IR \cdot u - k_2 \cdot IR_{ins} - k_{m,1} \cdot IR_{ins}$
$IR'_P = k_2 \cdot IR_{ins} + k_{1,basal} \cdot IR - k_3 \cdot IR_P + k_{m,3} \cdot IR_{iP} - k_{m,2} \cdot IR_P$
$IR'_{iP} = k_3 \cdot IR_P - k_{m,3} \cdot IR_{iP} - k_D \cdot IR_{iP}$
$IR'_i = k_D \cdot IR_{iP} - k_R \cdot IR_i$
$IRS' = -k_4 \cdot (IR_P + IR_{iP}) \cdot IRS + k_{m,4} \cdot IRS_P$
$IRS'_P = k_4 \cdot (IR_P + IR_{iP}) \cdot IRS - k_{m,4} \cdot IRS_P$
$y_A = k_{Y,A} \cdot (IR_P + IR_{iP})$
$y_B = k_{Y,B} \cdot IRS_P$
$y_C = k_{Y,C} \cdot IRS_P$
$y_D = k_{Y,D} \cdot IRS_P$

Table 8: States

State	Description
IR	Insulin receptor
IR_P	Phosphorylated insulin receptor
IR_{iP}	Internalized and phosphorylated insulin receptor
IR_i	Internalized and dephosphorylated bot not yet recycled insulin receptor
IR_{ins}	Insulin bound to the insulin receptor
IRS	Insulin receptor substrate
IRS_P	Phosphorylated insulin receptor substrate

Table 9: Parameters

Parameter	Description
k_i, $i \in \{1,2,3,4\}$	Rate constants
$k_{m,i}$, $i \in \{1,2,3,4\}$	Rate constants
k_D	Rate constant
$k_{1,basal}$	Rate constant
k_R	Rate constant
$k_{Y,j}$, $j \in \{A,B,C,D\}$	Scaling constants in measurement equations

Table 10: Input and Outputs

Variable	Description
u	Insulin administered
y_A	Output
y_B	Output
y_C	Output
y_D	Output

1.4 Model of NF-κB regulatory module [4]

$x_1' = -k_1x_1x_2 + \frac{1}{333}(-k_{14}x_1 + k_{15}x_9)$
$x_2' = -k_1x_1x_2 + \frac{1}{333}k_{13}x_8$
$x_3' = k_1x_2x_2 - \frac{1}{333}k_{11}x_3$
$x_4' = k_3 + k_2x_2 - k_4x_4$
$x_5' = k_6 + k_5x_2 - k_7x_5$
$x_6' = k_9 + k_8x_2 - k_{10}x_6$
$x_7' = \frac{10}{16667}k_{11}x_3 - k_{21}x_7 + k_1x_8x_9 - k_{28}x_7x_{11}$
$x_8' = k_{21}x_7 - \frac{10}{16667}k_{13}x_8 - k_1x_8x_9 + k_{26}x_{15}$
$x_9' = k_{18}x_5 - k_{23}x_9 - k_1x_8x_9 + \frac{10}{16667}(k_{14}x_1 - k_{15}x_9) - k_{25}x_9x_{11}$
$x_{10}' = k_{27}x_4 - k_{24}x_{10}$
$x_{11}' = -k_{12}x_{11} - k_{16}x_{11} - k_{28}x_7x_{11} - k_{25}x_9x_{11} - k_{19}x_{10}x_{11} + k_{17}x_{13} + k_{22}x_{14} + k_{26}x_{15}$
$x_{12}' = k_{16}x_{11} + k_{19}x_{10}x_{11} - k_{12}x_{12}$
$x_{13}' = k_{25}x_9x_{11} - k_{17}x_{13}$
$x_{14}' = k_{20} - k_{12}x_{14} - k_{22}x_{14}$
$x_{15}' = k_{28}x_7x_{11} - k_{26}x_{15}$
$y_1 = x_4$
$y_2 = x_5$
$y_3 = x_6$
$y_4 = x_{10}$

Table 11: States

Lipniacki	Our Equations	Description
$I\kappa B\alpha_n$	x_1	Molar concentration of nuclear $I\kappa B\alpha$
$NF\kappa B_n$	x_2	Molar concentration of free nuclear NF-κB
$(I\kappa B\alpha_n \vert NF\kappa B_n)$	x_3	Molar concentration of nuclear $(I\kappa B\alpha \vert NF$-$\kappa B)$ complexes
$A20_t$	x_4	Molar concentration of cytoplasmic mRNA transcript of $A20$
$I\kappa B\alpha_t$	x_5	Molar concentration of nuclear $I\kappa B\alpha$ transcript
$cgen_t$	x_6	Molar concentration of mRNA control gene transcript
$(I\kappa B\alpha \vert NF\kappa B)$	x_7	Molar concentration of cytoplasmic $I\kappa B\alpha \vert NF$-κB complexes
$NF\kappa B$	x_8	Molar concentration of free cytoplasmic NF-κB
$I\kappa B\alpha$	x_9	Molar concentration of free cytoplasmic $I\kappa B\alpha$ protein
$A20$	x_{10}	Molar concentration of cytoplasmic $A20$ protein
$IKKa$	x_{11}	Molar concentration of cytoplasmic IKK in the active state $IKKa$
$IKKi$	x_{12}	Molar concentration of cytoplasmic IKK in the inactive state $IKKi$
$(IKKa \vert I\kappa B\alpha)$	x_{13}	Molar concentration of cytoplasmic $IKKa \vert I\kappa B\alpha$ complexes
$IKKn$	x_{14}	Molar concentration of cytoplasmic IKK in the neutral state $IKKn$
$(IKKa \vert I\kappa B\alpha \vert NF\kappa B)$	x_{15}	Molar concentration of cytoplasmic $(IKKa \vert I\kappa B\alpha \vert NF$-$\kappa B)$ complexes

Table 12: Parameters

Lipniacki	Our Equations	Description
a_1	k_1	$I\kappa B\alpha$-$NF\kappa B$ association
c_1	k_2	$A20$-inducible mRNA synthesis
c_2	k_3	$A20$-constitutive mRNA synthesis
c_3	k_4	$A20$ mRNA degradation
c_{1a}	k_5	$I\kappa B\alpha$-inducible mRNA synthesis
c_{2a}	k_6	$I\kappa B\alpha$-constitutive mRNA synthesis
c_{3a}	k_7	$I\kappa B\alpha$ mRNA degradation
c_{1c}	k_8	cgen inducible mRNA synthesis
c_{2c}	k_9	cgen constitutive mRNA synthesis
c_{3c}	k_{10}	cgen mRNA degradation
e_{2a}	k_{11}	$(I\kappa B\alpha\|NF$-$\kappa B)$ nuclear export
k_{deg}	k_{12}	$IKKa$,$IKKn$ and $IKKi$ degradation
i_1	k_{13}	NF-κB nuclear import
e_{1a}	k_{14}	$I\kappa B\alpha$ nuclear export
i_{1a}	k_{15}	$I\kappa B\alpha$ nuclear import
k_3	k_{16}	IKK spontaneous activation rate
t_1	k_{17}	$IKKa\|I\kappa B\alpha$ catalysis
c_{4a}	k_{18}	$I\kappa B\alpha$ translation rate
$T_R k_2^*$	k_{19}	IKK activation rate caused by $A20$
k_{prod}	k_{20}	$IKKn$ production rate
c_{6a}	k_{21}	$I\kappa B\alpha$ degradation (complexed to NF-κB)
$T_R k_1^*$	k_{22}	IKK activation rate caused by TNF
c_{5a}	k_{23}	Spontaneous, free $I\kappa B\alpha$ protein degradation
c_5	k_{24}	$A20$ protein degradation
a_2	k_{25}	$IKKa$-$I\kappa B\alpha$ association
t_2	k_{26}	$(IKK\|I\kappa B\alpha\|NF$-$\kappa B)$ catalysis
c_4	k_{27}	$A20$ translation rate
a_3	k_{28}	$IKKa$-$(I\kappa B\alpha\|NF$-$\kappa B)$ association

* T_R is a binary signal, modulated by whether a signal is present ($T_R = 1$) or not ($T_R = 0$).

Table 13: Input and Output

Variable	Description
y_1	Output
y_2	Output
y_3	Output
y_4	Output

Table 14: Known Parameters

Known Parameter	Our Equations	Description
k_v	$\frac{1}{333}$	Cytoplasmic to nuclear volume
	$\frac{10}{16667}$	Volume-correction factor

References

[1] Cecilia Brännmark et al. "Mass and Information Feedbacks through Receptor Endocytosis Govern Insulin Signaling as Revealed Using a Parameter-free Modeling Framework". In: *Journal of Biological Chemistry* 285.26 (2010), pp. 20171–20179. URL: https://doi.org/10.1074/jbc.M110.106849.

[2] Belén Casas et al. "Integrated experimental-computational analysis of a liver-islet microphysiological system for human-centric diabetes research". In: *bioRxiv* (2021). URL: https://doi.org/10.1101/2021.08.18.456693.

[3] Albert Goldbeter. "A model for circadian oscillations in the Drosophila period protein (PER)". In: *Proceedings of the Royal Society of London. Series B: Biological Sciences* 261.1362 (1995), pp. 319–324. URL: http://doi.org/10.1098/rspb.1995.0153.

[4] Tomasz Lipniacki et al. "Mathematical model of NF-κB regulatory module". In: *Journal of Theoretical Biology* 228.2 (2004), pp. 195–215. ISSN: 0022-5193. URL: https://doi.org/10.1016/j.jtbi.2004.01.001.
